# Novel Silver Complexes Based on Phosphanes and Ester Derivatives of Bis(pyrazol-1-yl)acetate Ligands Targeting TrxR: New Promising Chemotherapeutic Tools Relevant to SCLC Management

**DOI:** 10.3390/ijms24044091

**Published:** 2023-02-17

**Authors:** Maura Pellei, Carlo Santini, Luca Bagnarelli, Miriam Caviglia, Paolo Sgarbossa, Michele De Franco, Mirella Zancato, Cristina Marzano, Valentina Gandin

**Affiliations:** 1 School of Science and Technology, Chemistry Division, University of Camerino, Via S. Agostino 1, 62032 Camerino, Italy; 2 Department of Industrial Engineering, University of Padova, Via Marzolo 9, 35131 Padova, Italy; 3 Department of Pharmaceutical and Pharmacological Sciences, University of Padova, Via Marzolo 5, 35131 Padova, Italy

**Keywords:** silver, bis(pyrazolyl)acetate ligands, anticancer activity, TrxR, oxidative stress

## Abstract

Bis(pyrazol-1-yl)acetic acid (HC(pz)_2_COOH) and bis(3,5-dimethyl-pyrazol-1-yl)acetic acid (HC(pz^Me2^)_2_COOH) were converted into the methyl ester derivatives **1** (L^OMe^) and **2** (L^2OMe^), respectively, and were used for the preparation of silver(I) complexes **3**–**5**. The Ag(I) complexes were prepared by the reaction of AgNO_3_ and 1,3,5-triaza-7-phosphaadamantane (PTA) or triphenylphosphine (PPh_3_) with L^OMe^ and L^2OMe^ in methanol solution. All Ag(I) complexes showed a significant in vitro antitumor activity, proving to be more effective than the reference drug cisplatin in the in-house human cancer cell line panel containing examples of different solid tumors. Compounds were particularly effective against the highly aggressive and intrinsically resistant human small-cell lung carcinoma (SCLC) cells, either in 2D and 3D cancer cell models. Mechanistic studies revealed their ability to accumulate into cancer cells and to selectively target Thioredoxin (TrxR), thus leading to redox homeostasis unbalance and ultimately inducing cancer cell death through apoptosis.

## 1. Introduction

Medicinal inorganic chemistry offers possibilities for the design of therapeutic agents not readily available to organic compounds [1,2]. The wide range of coordination numbers and geometries, accessible redox states, thermodynamic and kinetic characteristics, and the intrinsic properties of the cationic metal ion and ligand itself offer to the medicinal chemist a wide spectrum of reactivities that can be exploited [3]. Group 11 metal complexes showed encouraging perspectives in this regard, and several Cu(I), Cu(II), Au(I), Au(III) and Ag(I) complexes were investigated for their antitumoral properties [4,5,6,7,8]. In particular, silver(I) coordination compounds have been recognized as promising therapeutics [9] due to their outstanding antibacterial, antimycotic, antiparasitic, antimalarial and anticancer activities [4,10,11]. Silver(I) complexes for therapeutic purposes have been prepared with a vast variety of ligands, but those scoring the best results usually contain N-heterocycles, N-heterocyclic carbenes (NHC), and phosphanes [4,12,13,14,15,16,17]. Silver(I) coordination compounds are effective against various bacteria, fungi, protozoa, and several cancer cell lines, evidencing in the latter case anticancer mechanisms that share the same biological pathways as the antibacterial ones and for these reasons are completely different from those at the basis of cisplatin cytotoxicity [18,19]. To date and to the best of our knowledge, only a few studies have been reported on the effects of silver(I) complexes on tumor xenograft murine models, resulting in decreased growth of the tumor mass or in cell death [20,21,22]. Ag(I) compounds containing NHC ligands are the most promising in this respect [13,23,24].

The cytotoxic mechanisms of silver ions are based on a series of damages caused by Ag^+^ to the bacterial or cancer cells [4]. Nevertheless, nearly nothing is known about possible side effects that could develop after human administration of Ag(I) complexes, although silver is still considered to be nontoxic for humans and other mammalians.

The efficacy of silver(I) complexes against bacteria and cancer cells depends on a number of factors: lipophilicity, redox proclivity, water solubility and stability, and rate of release of the silver ions [25]. These factors are strictly controlled by the characteristics of the ligands and their requirements in both steric and electronic properties.

In this context, bis(azol-1-yl)acetate heteroscorpionate ligands, of the general formula [HC(CO_2_H)(az)_2_] (az = N-heterocyclic ring) [26,27,28], have recently attracted considerable attention, and their coordination chemistry toward main group and transition metals has been extensively studied [29,30]. Among them, complexes containing bis(pyrazol-1-yl)carboxylic acids are especially of interest, due to their *κ*^3^-N,N,O tripodal coordination behavior, as metalloenzyme models relevant for biochemistry [31,32,33,34,35] and as starting materials to yield bifunctional ligands [36,37,38,39,40].

We have recently reported that Cu(I) and Cu(II) complexes with heteroscorpionate ligands, obtained by conjugation of bis(pyrazolyl)acetates with nitroimidazole, glucosamine, a non-competitive NMDA receptor antagonist and the antineoplastic drug lonidamine showed cytotoxic activity toward a panel of several human tumor cell lines [36,37,38,39,41]. We have also investigated the capability of ester derivatives (methyl, isopropyl and hexyl) of bis(pyrazolyl)acetates species to form copper complexes, with in vitro antitumor activity [42], that are useful as efficient catalysts for allylic oxidations and organic macro(molecules) synthesis [43,44,45].

Therefore, the aim of this work was to study the capability of smaller ester derivatives of bis(pyrazolyl)acetates species to form Ag(I) complexes potentially useful for anticancer purposes. Bis(pyrazol-1-yl)acetic acid and bis(3,5-dimethyl-pyrazol-1-yl)acetic acid were converted into the methyl ester derivatives **1** (L^OMe^) and **2** (L^2OMe^) of bis(pyrazol-1-yl)- and bis(3,5-dimethyl-pyrazol-1-yl)-acetic acid, respectively, and used for the preparation of the Ag(I) complexes **3**–**5**. The lipophilic PPh_3_ and the hydrophilic PTA, able to stabilize silver in +1 oxidation state, were selected as coligands to confer different solubility properties to the resulting complexes. The new Ag(I) complexes **3**–**5** and the corresponding uncoordinated ligands (**1** and **2**) were investigated for their cytotoxic activity on a panel of human cancer cell lines, derived from different solid tumors, by means of both 2D and 3D cell viability studies. Mechanistic studies were performed with the aim to elucidate the molecular determinants accounting for their in vitro anticancer activity and to shed light onto the cell death mechanism triggered in SCLC cells, which were proven to be highly sensitive to their cytotoxic effects.

## 2. Results and Discussion

### 2.1. Synthesis and Characterization

The ligands [HC(pz)_2_COOCH_3_] (L^OMe^, **1**) and [HC(pz^Me2^)_2_COOCH_3_] (L^2OMe^, **2**) were prepared by a method described in the literature and were fully characterized. 

**Scheme 1:**
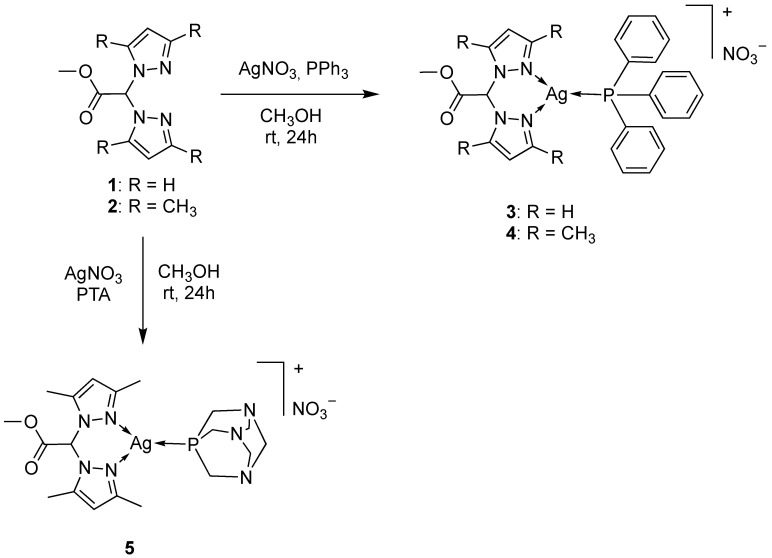
Chemical structures of complexes **3**–**5**.

The Ag(I) complexes [Ag(PPh_3_)(L^OMe^)]NO_3_ (**3**) and [Ag(PPh_3_)(L^2OMe^)]NO_3_ (**4**) were prepared from the reaction of PPh_3_, AgNO_3_ and the ligand L^OMe^ and L^2OMe^, respectively, following a one-pot synthesis in methanol as solvent. Analogously, the Ag(I) complex [Ag(PTA)(L^2OMe^)]NO_3_ (**5**) was prepared from the reaction of PTA, AgNO_3_ and the related ligand L^2OMe^ (Scheme 1). Attempts to synthesize the analogous compound using as starting materials AgNO_3_, PTA and L^OMe^ were unsuccessful, also using different solvents and different stochiometric ratios between the reagents.

All the compounds are soluble in CH_3_OH, CH_3_CN and dimethyl sulfoxide (DMSO); complexes **3** and **4** are also soluble in CHCl_3_. The conductivity measurements for complexes **3**–**5**, containing weakly coordinated NO_3_^−^ anion, are in accordance with the ionic formulations [46,47]. In particular, the molar conductance Λ_0_ for compounds **3** and **4** recorded in CH_3_CN is in the range 134–135 Ω^−1^ cm^2^ mol^−1^, in accordance with the presence of 1:1 electrolytes. The same behavior is observed for compound **5**, with a Λ_0_ = 25 Ω^−1^ cm^2^ mol^−1^ in DMSO solvent at 293 K. The IR spectra carried out on solid samples of the Ag(I) complexes showed all the expected bands for the chelating ligand and the phosphane coligand. The absorptions due to the C=O stretching of the ester groups are at 1760 cm^−1^ and they do not significantly vary with respect to the free ligands. In the region 2849–3137 cm^−1^, the complexes exhibit weak bands typical of C-H stretchings, while the NO_3_ vibrations appear as strong bands in the 1264–1380 cm^−1^ region. In the far-IR spectra, a number of weak to medium bands in the region 200–300 cm^−1^ absent in the spectra of the free ligands and of the AgNO_3_ can be assigned to Ag-N and Ag-P vibrations, in accordance with previous assignments [48]. The ^1^H-NMR spectra of the Ag(I) complexes, recorded in CD_3_CN solution at room temperature, showed a single set of resonances for the pyrazole rings, indicating that the pyrazole protons are equivalent, with a slight shift due to the coordination to the metal center. The PPh_3_ and PTA coligands showed a characteristic series of peaks at δ 7.46–7.60 and 4.35–4.67 ppm, respectively, with an integration that confirms the 1:1 stoichiometric ratio between the ligand and the phosphane coligand. The room temperature ^31^P{H}-NMR spectra of the Ag(I) complexes, recorded in CD_3_CN solution at room temperature, gave signals downfield shifted with respect to the value of the free phosphanes PPh_3_ and PTA (δ = −4.85 and −102.07 ppm, respectively). In particular, the room temperature spectrum of **3** in CD_3_CN gave a broad singlet centered at δ 10.67, while **4** exhibits a broad doublet with a ^1^*J*(Ag-^31^P) coupling constant of 626 Hz. At 243 K, the spectra of **3** and **4** (in CD_3_CN solvent) show two doublets in which the coupling of ^31^P to the ^107^Ag and ^109^Ag are resolved, in accordance with a stopped or slow phosphane exchange process. The ^1^*J*(^107^Ag-^31^P) and ^1^*J*(^109^Ag-^31^P) coupling constants are respectively in the range 611–624 and 705–721 Hz for compounds **3** and **4**, being of the same order of magnitude of those reported for analogous silver(I) monophosphane species [48,49,50]. The room temperature spectrum of **5** in CD_3_CN gave a broad singlet centered at δ –84.87, while at 243 K (in CD_3_CN solvent), it exhibits two doublets revealing the coupling of the two silver isotopes (^1^*J*(^107^Ag-^31^P) = 614 Hz and ^1^*J*(^109^Ag-^31^P) = 694 Hz), in the same order of magnitude of analogous silver(I) monophosphane species. The ratio of ^1^*J*(^109^Ag-^31^P)/^1^*J*(^107^Ag-^31^P) is in good agreement with the ^107^Ag/^109^Ag gyromagnetic ratio of 1.15.

The ESI-MS studies, performed by dissolving the Ag(I) complexes in CH_3_CN and recording the spectra in positive- and negative-ion mode, confirmed the formation of the PPh_3_ and PTA complexes and the presence of nitrates as counterions. In particular, the formation of complexes **3**–**5** was confirmed by the presence in the positive-ion ESI-MS spectra of the major peaks attributable to the [Ag(PPh_3_)(L^OMe^)]^+^, [Ag(PPh_3_)(L^2OMe^)]^+^ and [Ag(PTA)(L^2OMe^)]^+^ species, respectively. In the negative-ion spectra, [Ag(NO_3_)_2_]^−^ was observed as the major peak for all the complexes. The elemental analyses confirmed the stoichiometry and the purity of the products.

### 2.2. Cytotoxicity Studies

The Ag(I) complexes **3**–**5** and the corresponding uncoordinated ligands **1** and **2** were evaluated for their cytotoxic activity against various human cancer cell lines representative of different solid tumors. In particular, the in-house cancer cell panel contained examples of human colon (HCT-15), pancreatic (PSN-1), cervical (A431), breast (MDA-MB-231) and ovarian (2008) carcinoma as well as of SCLC (U1285). The cytotoxicity parameters, expressed in terms of IC_50_ obtained after 72 h of exposure to the MTT assay, are reported in Table 1. For comparison purposes, the cytotoxicity of the reference metal-based chemotherapeutic drug cisplatin was assessed under the same experimental conditions.

The three Ag(I) complexes demonstrated a marked cytotoxic activity against all tested cell lines, showing IC_50_ values in the low/sub micromolar range, and being on average more effective than cisplatin. In particular, complexes bearing the L^2OMe^ ligand (**4** and **5**) were the most effective derivatives, with average IC_50_ values of 4.0 and 3.0 µM, respectively. Conversely, the L^OMe^-bearing derivative **3** was the weakest of the series in decreasing cancer cell viability (average IC_50_ value of 7.5 µM), although it retained a cytotoxicity profile better than that of cisplatin against HCT-115, MDA-MB-231 and U1285 cancer cell lines. Bidimensional in vitro cytotoxicity screening highlighted human SCLC cells as the most sensitive to the cytotoxic effect induced by newly developed Ag(I) complexes. Actually, complexes **3**–**5** were from 5 up to 14 times more effective than cisplatin in decreasing U1285 cancer cell viability.

The antiproliferative activity of the Ag(I) complexes **3**–**5** was also investigated in an additional cell line selected for its resistance to cisplatin, the human ovarian adenocarcinoma C13* cells. These cells were suitably selected after a cisplatin chronic treatment and are characterized by several mechanisms accounting for their resistance to cisplatin; these include (i) a reduced intracellular drug accumulation [51], (ii) high cellular levels of glutathione and metallothioneins [52], (iii) the enhanced repair of platinum-DNA adducts [53], and (iv) an overexpression of the Trx system [54].

Cytotoxicity was assessed after 72 h of drug treatment by MTT test, and the IC_50_ and RF values (RF = resistance factor, defined as the ratio of the IC_50_ of resistant cells over the IC_50_ of sensitive ones) are reported in Table 1. All new Ag(I) complexes exhibited a similar cytotoxic potency both on sensitive and resistant cells, thus proving to be capable of overcoming cisplatin resistance. Again, complexes **4** and **5** bearing the L^2OMe^ ligand were the most active compounds, being able to elicit IC_50_ values in the sub-micromolar range.

One of the main drawbacks of chemotherapeutic drugs is the possible toxic effect toward non-cancerous cells, and we measured the cytotoxicity of Ag(I) complexes against non-cancerous HEK293 cells (see Table 1). For all Ag(I) complexes, calculated IC_50_ values were higher than those obtained on average against tumor cells, thus indicating for this class of metal complexes a slightly preferential cytotoxicity against human cancer cells. In particular, the selectivity index values (SI = the quotient of the average IC_50_ toward normal cells divided by the average IC_50_ for the malignant cells) of **3**–**5** were 2.3, 3.9 and 4.6, respectively.

The in vitro antitumor activity of the newly developed Ag(I) derivatives was also assayed in 3D cell culture models of SCLC cells. Even if the 2D cell cultures are the most employed assays for in vitro screening due to the low cost, simplicity and reliability, two-dimensional methods are unable to reproduce the properties of in vivo solid tumors. In contrast, 3D cell cultures are much more efficient in closely mimicking the heterogeneity and complexity of the tumor mass and therefore are more predictive for in vivo results than conventional 2D cell cultures [55]. On these bases, we tested the activity of the Ag(I) complexes on spheroids obtained from U1285 cells, which were proven to be highly sensitive to their in vitro antitumor activity. SCLC spheroids were treated with the investigated compounds for 72 h, and cell viability was assessed by means of the acid phosphatase (APH) assay.

Results reported in Table 2 clearly attest that all three Ag(I) complexes were more effective than the reference drug cisplatin against 3D spheroids, being again compound **4** and **5** bearing the L^2OMe^ ligand as the most active ones. In particular, compounds **4** and **5** showed IC_50_ values averagely 2.5 times lower than that calculated for **3** and cisplatin.

Spheroids from U1285 cells (3 × 10^3^ cells/well) were treated for 72 h with increasing concentrations of tested compounds. The growth inhibitory effect was evaluated by means of the APH assay. IC_50_ values were calculated from the dose–survival curves by the four-parameter logistic model (*p* < 0.05). S.D. = standard deviation.

Altogether, 2D and 3D cytotoxicity results suggested that the L^2OMe^-bearing Ag(I) complexes were much more effective in entering cancer cells or in penetrating across the entire spheroid domain.

### 2.3. Cellular Uptake and Mechanistic Studies

**Figure 1:**
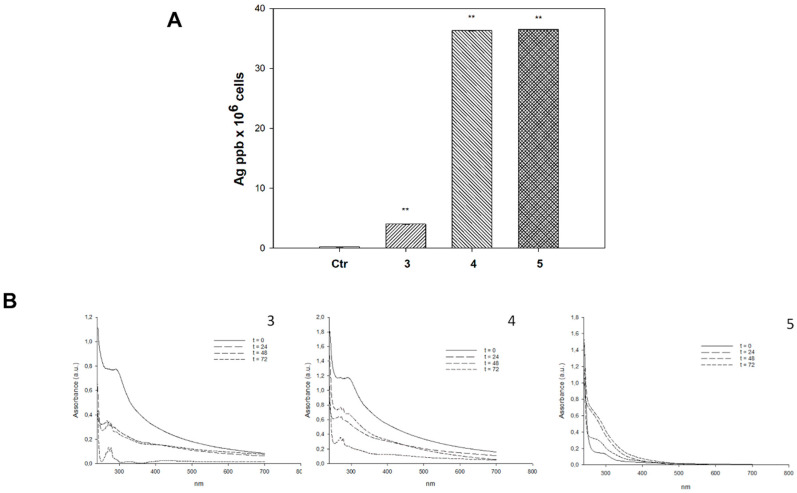
Cellular uptake and stability studies. (**A**) Intracellular accumulation of silver(I) complexes **3**–**5**. U1285 cells were incubated with 2 μM of silver(I) complexes for 24 h, and cellular silver content was detected by GF-AAS analysis. Error bars indicate the standard deviation. ** *p* < 0.01 compared with control. (**B**) Stability studies. All complexes were dissolved at 50 μM in 0.5% DMSO/RPMI. UV–visible spectra were recorded at t = 0 min, t = 24 h, t = 48 h and t = 72 h.

To demonstrate the soundness of the above-reported supposition, we investigated the ability of tested complexes to accumulate into human U1285 cancer cells. Cells were treated for 24 h with equimolar concentrations of **3**–**5** (2 µM), and silver content was quantified using graphite furnace atomic absorption spectrometry (GF-AAS) analysis. The results, expressed as ppm of metal per 10^6^ cells and depicted in Figure 1, clearly confirm that the most effective derivatives **4** and **5** were those that were more effectively accumulated into human lung cancer cells. However, no linear correlation between antiproliferative activity and cellular uptake as well as between cellular uptake and logP values of the phoshane ligand could be clearly shown (see Figure S1), thus corroborating the hypothesis that lipophilicity of phosphane coligand only marginally contributes to the Ag(I) complexes uptake and efficacy profiles. On the contrary, the lipophilicity of the bidentate bis(pyrazol-1-yl)acetate ligands seems to highly contribute to their in vitro biological features, being the complexes bearing the more lipophilic L^2OMe^ ligand much more effective than those bearing the L^OMe^ one.

Additionally, stability studies performed in 0.5% DMSO/RPMI cell culture medium using UV−vis spectroscopy showed that complexes bearing the PPh_3_ ligand were endowed with a scarce stability profile. Actually, significant changes were observed in the UV−vis spectra of Ag(I) complexes. This feature does not seem to affect their biological activity. Our results showing that under physiological conditions Ag(I), complex spectra rapidly (within 24 h) changed, presumably attesting the quick formation of the metal species responsible for the antiproliferative activity.

Ag(I) complexes have been widely described to be effective inhibitors of redox-active Sec-containing Thioredoxin reductase [56,57,58]. In addition, SCLC is endowed with elevated TrxR/Trx levels, which are known to account for radio- and chemo-resistance [59,60].

**Figure 2:**
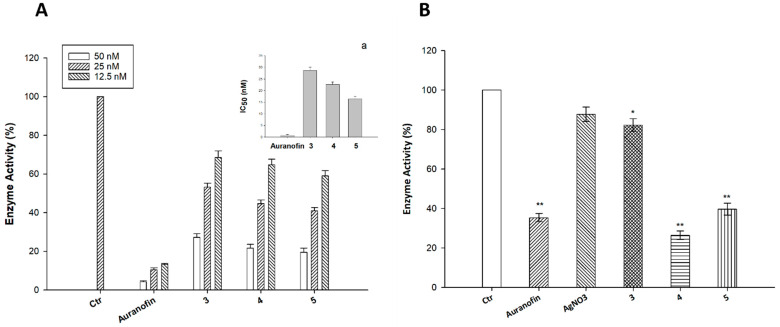
TrxR inhibition. (**A**) TrxR1 activity was assayed by measuring NADPH-dependent reduction of DTNB at 412 nm as described in the Experimental Section. (a) 50% inhibition concentration (IC_50_), Error bars indicate S.D. (**B**) U1285 cells were incubated for 24 h with the tested compounds (2 µM). Subsequently, cells were washed twice with PBS and lysed. TrxR activity was tested by measuring NADPH-dependent reduction of DTNB at 412 nm. Error bars indicate SD. * *p* < 0.05; ** *p* < 0.01.

On these bases, we thought it of interest to evaluate the ability of compounds **3**–**5** to inhibit TrxR1 both in cell-free systems and in intact human U1285 cancer cells, and their activity was compared to that of auranofin, a well-known metal-based inhibitor of TrxR. Enzyme activity was measured according to standard procedures described in the Experimental Section, and results are shown in Figure 2A,B. In cell-free experiments, all three complexes proved to be strongly effective in inhibiting cytosolic mammalian TrxR1 in a dose-dependent manner (Figure 2A), showing IC_50_ values in the nanomolar range (Figure 2A, insert a). However, their efficacy in in vitro experiments was lower than that shown by the reference TrxR inhibitor auranofin.

On the other hand, Ag(I) compounds **4** and **5** were as effective or even better than auranofin in hampering TrxR activity when tested in U1285 cell cultures. Actually, derivative **4** tested at 2 µM was able to decrease TrxR enzyme activity by about 68%, compared with auranofin, which at the same concentration, induced a 62% inhibition of the Sec-containing redox enzyme (Figure 2B). These results clearly confirmed the ability of Ag(I) complexes to target TrxR in intact cancer cells. 

It is widely known that Trx system plays an essential role in cellular redox homeostasis, and inhibition of this redox regulatory system has been shown to determine cellular redox unbalance in terms of sulfhydryl redox status and cellular production of reactive oxygen species (ROS) [61,62,63]. 

**Figure 3:**
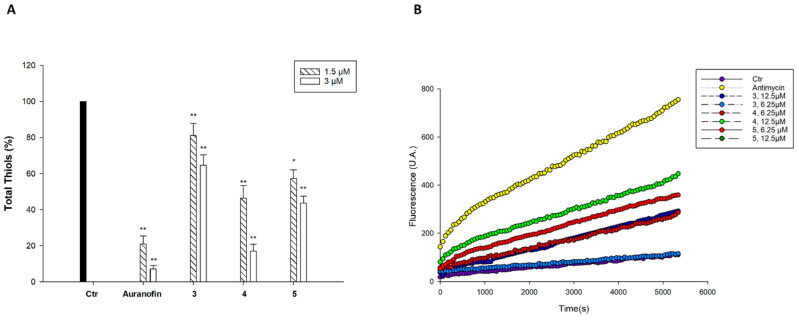
Effects on sulfhydryl content and ROS production. (**A**) Sulfhydryl content in U1285-treated cancer cells incubated for 24 h with tested compounds. The sulfhydryl group amount was determined by the DTNB assay. Error bars indicate S.D. * *p* < 0.05, ** *p* < 0.01. (**B**) Effect of silver(I) compounds on hydrogen peroxide formation in U1285 cells. Cells were pre-incubated in PBS/10 mM glucose medium for 20 min at 37 °C in the presence of 10 μM CM–DCFDA and then treated with increasing concentrations of the tested compounds.

Hence, the effect induced by the newly developed heteroleptic Ag(I) complexes on total cellular sulfhydryl content and on ROS production was assayed in U1285-treated cells. As evident in Figure 3A, although to a different extent, all three silver(I) complexes were effective in modulating total thiol content of U1285 cells in a dose-dependent manner. In particular, the reduction of cellular sulfhydryl content obtained with **4** at the higher concentration tested (3 µM) was very similar to that induced by equidoses of auranofin.

Consistently, treatment of U1285 cells with Ag(I) complexes determined a substantial time-dependent and dose-dependent increase in cellular basal hydrogen peroxide production (Figure 3B), which was, however, less pronounced with respect to that elicited by antimycin, a classic inhibitor of the mitochondrial respiratory chain at the level of complex III.

Mitochondria are considered the main cellular source of ROS, and the magnitude of a change in mitochondrial ROS production strictly depends on the physicochemical state of this organelle. A persistent increase in the rate of ROS production and the induction of thiol redox stress are consistent with the collapse of mitochondrial shape and integrity (swelling), ultimately leading to the induction of cell apoptosis [64]. We therefore evaluated the effect determined by treatment with our newly synthesized Ag(I) complexes in terms of modification of mitochondrial pathophysiological characteristics, such as mitochondrial membrane potential and morphological changes, in SCLC cells. 

**Figure 4:**
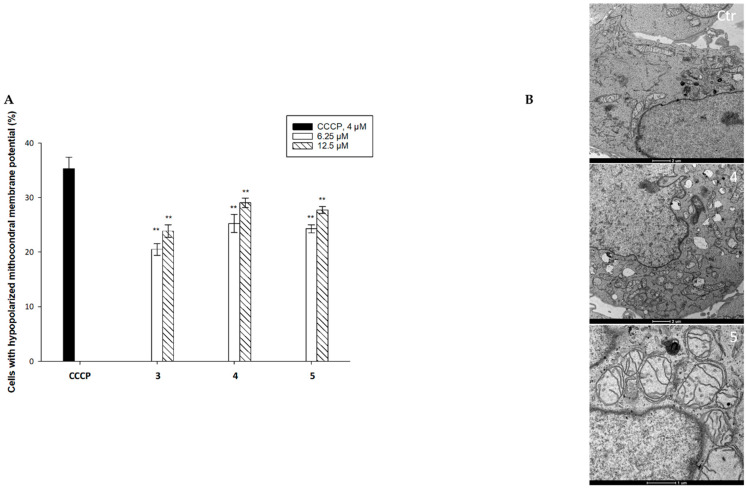
Antimitochondrial effect. (**A**) U1285 cells were treated for 24 h with increasing concentrations of the tested complexes or CCCP (3 μM). The percentage of cells with hypopolarized mitochondrial membrane potential was determined by Mito-ID^®^ Membrane Potential Kit. ** *p* ≤ 0.01. Error bars indicate S.D. (**B**) TEM analysis. Transmission electron micrographs of untreated U1285 cells or 24 h treated cells with IC_50_ concentrations of complex **3** or complex **4**.

For MMP detection, U1285 cells were treated with increasing concentrations of the tested complexes, and the percentage of cells with hypopolarized mitochondrial membrane potential was determined by means of the Mito-ID^®^ Membrane Potential Kit. The carbonyl cyanidem-chlorophenylhydrazone (CCCP) was used as positive control. Results depicted in Figure 4A show that the percentage of hypopolarized cells induced by treatment with tested complexes was dose-dependent and reached about 30% for compound **4** at the highest tested doses. Again, the L^2OMe^-bearing complexes **4** and **5** were those eliciting the highest effect, in accordance with all the above-reported results.

In agreement, TEM analyses performed on U1285 cells treated for 24 h with IC_50_ concentrations of the representative complexes **4** and **5** showed a dramatic swelling of the mitochondria, associated with a decrease in the electron density of the inner membrane and matrix regions, as clear signs of their antimitochondrial effect, thus confirming that they elicited a substantial modification of the mitochondria physiology (see Figure 4B).

**Figure 5:**
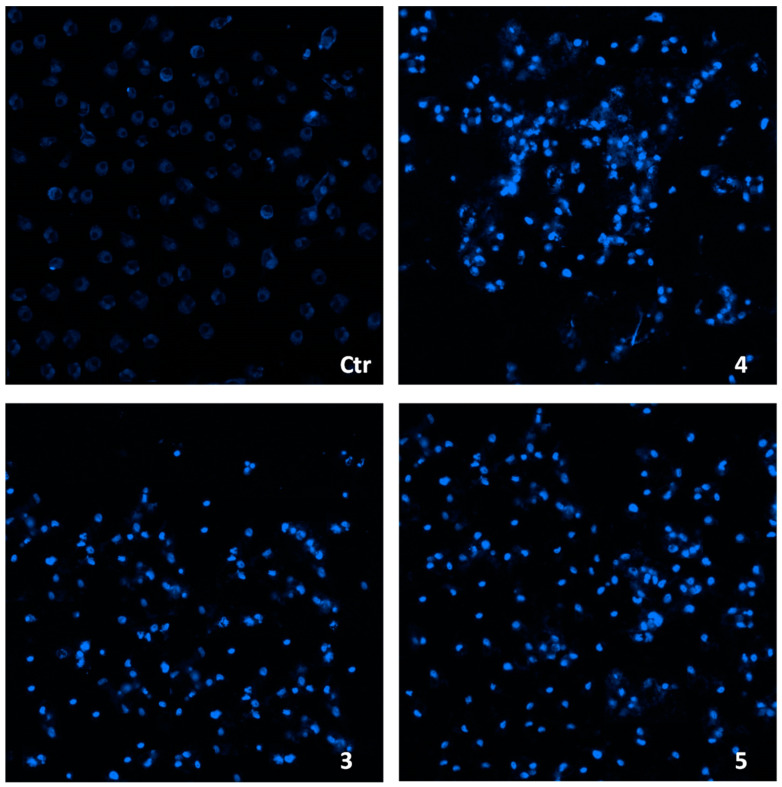
Hoechst staining (magnification, ×20) of U1285 cells incubated for 48 h with IC_50_ doses of **3**–**5**.

Finally, owing to the well-established connection between oxidative stress and apoptosis induction, we assessed the ability of tested complexes to induce cancer cell death by triggering apoptosis. Figure 5 shows the results obtained upon monitoring cellular morphological changes in U1285 cells treated for 48 h with IC_50_ doses of **3**–**5** and stained with Hoechst 33258 fluorescent probe. Compared with control cells, cells treated with the tested complexes presented brightly stained nuclei and morphological features typical of cells undergoing apoptosis, such as chromatin condensation, thus confirming the ability of the newly developed Ag(I) complexes to induce apoptotic cell death in U1285 cells. 

## 3. Materials and Methods

### 3.1. Chemistry

#### 3.1.1. Materials and General Methods

All reagents were purchased and used without further purification. All solvents were dried, degassed, and distilled prior to use. Melting points were performed by an SMP3 Stuart Scientific Instrument (Bibby Sterilin Ltd., London, UK). Elemental analyses (C, H, N, S) were performed with a Fisons Instruments EA-1108 CHNS-O Elemental Analyzer (Thermo Fisher Scientific Inc., Waltham, MA, USA). Fourier-transform infrared (FT-IR) spectra were recorded from 4000 to 200 cm^−1^ on a PerkinElmer Frontier Instrument (PerkinElmer Inc., Waltham, MA, USA), equipped with Attenuated Total Reflection (ATR) unit using universal diamond top-plate as sample holder. Abbreviation used in the analyses of the FT-IR spectra: m = medium, mbr = medium broad, s = strong, sbr = strong broad, sh = shoulder, vs = very strong, w = weak, wbr = weak broad. ^1^H-, ^13^C- and ^31^P-NMR spectra were recorded with a Bruker 500 Ascend Spectrometer (Bruker BioSpin Corporation, Billerica, MA, USA; 500.1 MHz for ^1^H, 125 MHz for ^13^C and 202,4 MHz for ^31^P). Referencing was relative to tetramethylsilane (TMS) (^1^H and ^13^C) and 85% H_3_PO_4_ (^31^P). NMR annotations used were as follows: br = broad, d = doublet, dbr = doublet broad, m = multiplet, mbr = multiplet broad, s = singlet, sbr = singlet broad, sept = septet, t = triplet. Electrospray ionization mass spectra (ESI-MS) were recorded in positive- (ESI-MS(+)) or negative-ions (ESI-MS(−)) mode on a Waters Micromass ZQ Spectrometer equipped with a single quadrupole (Waters Corporation, Milford, MA, USA), using a methanol or acetonitrile mobile phase. The compounds were added to reagent-grade methanol or acetonitrile to give approximately 0.1 mM solutions. These solutions were injected (1 µL) into the spectrometer fitted with an autosampler. The pump delivered the solutions to the mass spectrometer source at a flow rate of 200 μL/min, and nitrogen was employed both as a drying and nebulizing gas. Capillary voltage was typically 2500 V. The temperature of the source was 100 °C, while the temperature of the desolvation was 400 °C. In the analyses of ESI-MS spectra, the confirmation of major peaks was supported by comparison of the observed and predicted isotope distribution patterns, the latter calculated using the IsoPro 3.1 computer software (T-Tech Inc., Norcross, GA, USA). The electrical conductivity of the acetonitrile and dimethyl sulfoxide solutions was measured with a Crison CDTM 522 conductivity meter (Crison Instruments SA, Barcelona, Spain) at room temperature.

The precursors HC(pz)_2_COOH (LH) [27] and HC(pz^Me2^)_2_COOH (L^2^H) [26] were prepared by literature method. The ligands [HC(pz)_2_COOCH_3_] (L^OMe^, **1**) [65] and [HC(pz^Me2^)_2_COOCH_3_] (L^2OMe^, **2**) were prepared by literature method [66] and were fully characterized (^1^H-NMR: Figures S2 and S3).

#### 3.1.2. Synthesis of [Ag(PPh_3_)(L^OMe^)]NO_3_ (**3**)

AgNO_3_ (1.000 mmol, 0.170 g) was added to a solution of triphenylphosphine (PPh_3_, 1.000 mmol, 0.262 g) in methanol (30 mL). The reaction mixture was stirred at room temperature for 3 h; then, ligand **1** (1.000 mmol, 0.206 g) was added, and the solution was stirred for 24 h at room temperature in the dark. The solution was dried at reduced pressure, and the residue was recrystallized by diethyl ether to give the whitish complex **3** in 90% yield. M.p.: 165–168 °C. *Λ*_0_ (CH_3_CN, 10^−3^ M, 293 K): 134 Ω^−1^ cm^2^ mol^−1^. FT-IR (cm^−1^) (Figure S4): 3117wbr, 3058wbr, 2938wbr, 2886wbr, 2849wbr (C-H); 1760s (C=O); 1523wbr, 1480m, 1454m (C=C/C=N); 1436m, 1398s, 1379m; 1314s, 1299s, 1283s (NO_3_); 1257m, 1228s, 1207m, 1182m, 1159m, 1095s, 1062m, 1039m, 1027m, 999m, 983m, 972m, 919m, 900m, 851m, 825m, 810m, 763s, 748vs, 707m, 694s, 656m, 617m, 597m, 523s, 493s, 437w, 403w, 365w, 346w, 331m, 281w, 250w, 228w, 210m. ^1^H-NMR (CD_3_CN, 293 K, Figure S5): δ 3.69 (s, 3H, OC*H*_3_), 6.42 (t, ^3^*J*_HH_ = 6.4 Hz, 2H, 4-C*H*), 7.46-7.58 (m, 16H, C*H*CO and C*H*_ar_), 7.65 (d, ^3^*J*_HH_ = 7.7 Hz, 2H, 5-C*H*), 7.96 (d, ^3^*J*_HH_ = 8.0 Hz, 2H, 3-C*H*). ^1^H-NMR (CDCl_3_, 293 K): δ 3.75 (s, 3H, OC*H*_3_), 6.36 (t, ^3^*J*_HH_ = 6.4 Hz, 2H, 4-C*H*), 7.48-7.55 (m, 16H, C*H*CO and C*H*_ar_), 7.59 (d, ^3^*J*_HH_ = 7.6 Hz, 2H, 5-C*H*), 8.25 (d, ^3^*J*_HH_ = 8.3 Hz, 2H, 3-C*H*). ^13^C{^1^H}-NMR (CD_3_CN, 293 K, Figure S6): δ 53.46, 73.30, 106.99, 129.21, 129.29, 130.99, 131.01, 131.23, 131.97, 133.72, 141.86, 164.94. ^31^P{^1^H}-NMR (CD_3_CN, 293 K): δ 10.67 (br). ^31^P{^1^H}-NMR (CD_3_CN, 243 K, Figure S7): δ 10.79 (d, ^1^*J*(^107^Ag-^31^P) = 624 Hz and d, ^1^*J*(^109^Ag-^31^P) = 722 Hz). ^31^P{^1^H}-NMR (CDCl_3_, 293 K): δ 16.87 (d, ^1^*J*(Ag-^31^P) = 671 Hz). ^31^P{^1^H}-NMR (CDCl_3_, 243 K): δ 16.09 (d, ^1^*J*(^107^Ag-^31^P) = 662 Hz; d, ^1^*J*(^109^Ag-^31^P) = 749 Hz). ^31^P{^1^H}-NMR (CD_3_OD, 293 K): δ 13.95 (d, ^1^*J*(Ag-^31^P) = 723 Hz). ^31^P{^1^H}-NMR (CD_3_OD, 223 K, Figure S8): δ 12.01 (d, ^1^*J*(^107^Ag-^31^P) = 642 Hz; d, ^1^*J*(^109^Ag-^31^P) = 742 Hz). ESI-MS (major positive ions, CH_3_CN), *m*/*z* (%): 313 (20) [Ag(L^OMe^)]^+^, 371 (25) [Ag(PPh_3_)]^+^, 577 (100) [Ag(PPh_3_)(L^OMe^)]^+^, 633 (50) [Ag(PPh_3_)_2_]^+^. ESI-MS (major negative ions, CH_3_CN), *m*/*z* (%): 231 (100) [Ag(NO_3_)_2_]^−^. Elemental analysis (%) calculated for C_27_H_25_AgN_5_O_5_P: C 50.80, H 3.95, N 10.97; found: C 50.81, H 3.95, N 10.80.

#### 3.1.3. Synthesis of [Ag(PPh_3_)(L^2OMe^)]NO_3_ (**4**)

This compound was prepared following the procedure described for **3**, using ligand **2** (1.000 mmol, 0.262 g), to give the whitish complex **4** in 89% yield. M.p.: 188–193 °C. *Λ*_0_ (CH_3_CN, 10^−3^ M, 293 K): 135 Ω^−1^ cm^2^ mol^−1^. FT-IR (cm^−1^) (Figure S9): 3136wbr, 3051wbr, 2956wbr, 2921wbr (C-H); 1760s (C=O); 1561m, 1471mbr (C=C/C=N); 1436m, 1420m; 1380mbr, 1323sbr, 1299s, 1270m (NO_3_); 1221m, 1185m, 1154wbr, 1124wbr, 1112wbr, 1097m, 1073wbr, 1032mbr, 999mbr, 976sh, 918w, 891m, 870m, 824m, 803m, 782m, 757m, 743s, 720m, 694s, 639w, 603w, 523s, 503m, 488m, 447m, 422w, 391w, 367w, 281w, 269w, 235w, 224m, 204m. ^1^H-NMR (CD_3_CN, 293 K, Figure S10): δ 2.17 (s, 6H, 3- or 5-C*H*_3_), 2.46 (s, 6H, 3- or 5-C*H*_3_), 3.42 (s, 3H, OC*H*_3_), 6.12 (s, 2H, 4-C*H*), 7.04 (s, 1H, C*H*CO), 7.50-7.59 (m, 15H, C*H*_ar_). ^1^H-NMR (CDCl_3_, 293 K): δ 2.09 (s, 6H, 3-C*H*_3_), 2.55 (s, 6H, 5-C*H*_3_), 3.75 (s, 3H, OC*H*_3_), 5.97 (s, 2H, 4-C*H*), 7.49-7.51 (m, 16H, C*H*CO and C*H*_ar_). ^13^C{^1^H}-NMR (CD_3_CN, 293 K, Figure S11): δ 10.31, 13.53, 53.53, 66.60, 106.63, 129.25, 131.07, 131.55, 133.68, 143.50, 151.38, 164.96. ^31^P{^1^H}-NMR (CD_3_CN, 293 K): δ 12.74 (dbr, ^1^*J*(Ag-^31^P) = 626 Hz). ^31^P{^1^H}-NMR (CD_3_CN, 243 K, Figure S12): δ 11.26 (d, ^1^*J*(^107^Ag-^31^P) = 611 Hz; d, ^1^*J*(^109^Ag-^31^P) = 705 Hz). ^31^P{^1^H}-NMR (CDCl_3_, 293 K): δ 16.03 (dbr, ^1^*J*(Ag-^31^P) = 683 Hz). ^31^P{^1^H}-NMR (CDCl_3_, 223 K, Figure S13): δ 12.03 (d, ^1^*J*(^107^Ag-^31^P) = 628 Hz; d, ^1^*J*(^109^Ag-^31^P) = 725 Hz). ESI-MS(+) (major positive ions, CH_3_CN), *m*/*z* (%): 371 (15) [Ag(PPh_3_)]^+^, 633 (100) [Ag(PPh_3_)(L^2OMe^)]^+^. ESI-MS(-) (major negative ions, CH_3_CN), *m*/*z* (%): 231 (100) [Ag(NO_3_)_2_]^−^. Elemental analysis (%) calculated for C_31_H_33_AgN_5_O_5_P: C 53.61, H 4.79, N 10.08; found: C 53.68, H 4.84, N 9.93.

#### 3.1.4. Synthesis of [Ag(PTA)(L^2OMe^)]NO_3_ (**5**)

AgNO_3_ (1.000 mmol, 0.170 g) was added to a solution of 1,3,5-triaza-7-phosphaadamantane (PTA, 1.000 mmol, 0.157 g) in methanol (30 mL). The reaction mixture was stirred at room temperature for 3 h; then, ligand **2** (1.000 mmol, 0.262 g) was added, and the solution was stirred for 24 h at room temperature in the dark. The solution was dried at reduced pressure, and the residue was recrystallized by diethyl ether to give the whitish complex **5** in 78% yield. M.p.: 210–214 °C. *Λ*_0_ (DMSO, 10^−3^ M, 293 K): 25 Ω^−1^ cm^2^ mol^−1^. FT-IR (cm^−1^) (Figure S14): 3137wbr, 2958wbr, 2920wbr, 2852wbr (C-H); 1760m (C=O); 1642w; 1561w (C=C/C=N); 1447wbr, 1419m, 1395m, 1385m, 1367m; 1320mbr, 1296mbr (NO_3_); 1264m, 1241m; 1220m, 1155w, 1126w, 1107w, 1099w, 1036w, 1013mbr, 973m, 950m, 890w, 866w, 810m, 791m, 782mbr, 753wbr, 710m, 639m, 605m, 580m, 565m, 484m, 454m, 397m, 349m, 280m, 251m, 209m. ^1^H-NMR (CD_3_CN, 293 K, Figure S15): δ 2.20 (s, 6H, 3-C*H*_3_), 2.38 (s, 6H, 5-C*H*_3_), 3.78 (s, 3H, OC*H*_3_), 4.35 (sbr, 6H, NC*H*_2_P), 4.53-4.67 (m, 6H, NC*H*_2_N), 6.06 (s, 2H, 4-C*H*), 6.95 (s, 1H, C*H*CO). ^13^C{^1^H}-NMR (CD_3_CN, 293 K, Figure S16): δ 10.27, 13.37, 50.60, 53.61, 67.23, 72.57, 106.53, 143.15, 150.88, 164.91. ^31^P{^1^H}-NMR (CD_3_CN, 293 K): δ −84.87 (sbr). ^31^P{^1^H}-NMR (CD_3_CN, 243 K, Figure S17): δ −86.11 (d, ^1^*J*(^107^Ag-^31^P) = 609 Hz; d, ^1^*J*(^109^Ag-^31^P) = 698 Hz). ^31^P{^1^H}-NMR (CD_3_OD, 293 K): δ −82.11 (sbr). ^31^P{^1^H}-NMR (CD_3_OD, 223 K): δ −81.26 (d, ^1^*J*(^107^Ag-^31^P) = 624 Hz; d, ^1^*J*(^109^Ag-^31^P) = 698 Hz). ESI-MS(+) (major positive ions, CH_3_CN), *m*/*z* (%): 158 (20) [PTA + H]^+^, 264 (5) [Ag(PTA)]^+^, 371 (30) [Ag(L^2OMe^)]^+^, 528 (100) [Ag(PTA)(L^2OMe^)]^+^, 633 (50) [Ag(L^2OMe^)_2_]^+^. ESI-MS(-) (major negative ions, CH_3_CN), *m*/*z* (%): 62 (100) [NO_3_]^−^, 231 (10) [Ag(NO_3_)_2_]^−^. Elemental analysis (%) calculated for C_19_H_30_AgN_8_O_5_P:C 39.42, H 5.13, N 19.01; found: C 40.00, H 5.32, N 19.51.

#### 3.1.5. Stability Studies in DMSO/RPMI

All complexes were dissolved at 50 μM in 0.5% DMSO/RPMI. UV-Vis spectra were recorded at t = 0 min, t = 24 h, t = 48 and t = 72 h by a Perkin-Elmer Lambda 25 (Perkin-Elmer).

### 3.2. Experiments with Cultured Human Cancer Cells

Ag(I) complexes and the corresponding uncoordinated ligands were dissolved in DMSO just before the experiment, and a calculated amount of drug solution was added to the cell growth medium to a final solvent concentration of 0.5%, which had no detectable effects on cell viability. Cisplatin was dissolved in 0.9% sodium chloride solution. MTT (3-(4,5-dimethylthiazol-2-yl)-2,5-diphenyltetrazolium bromide) and cisplatin were obtained from Sigma Chemical Co, St. Louis, MO, USA.

#### 3.2.1. Cell Cultures

Human SCLC (U1285), breast (MDA-MB-231), colon (HCT-15), and pancreatic (PSN-1) carcinoma cell lines were obtained by American Type Culture Collection (ATCC, Rockville, MD, USA). A431 are human cervical carcinoma cells kindly provided by Professor F. Zunino (Division of Experimental Oncology B, Istituto Nazionale dei Tumori, Milan, Italy). The 2008 cells and the cisplatin-resistant variant, C13* cells, are human ovarian adenocarcinoma cell lines that were kindly provided by Professor G. Marverti (Department of Biomedical Science of Modena University, Modena, Italy). Cell lines were maintained in the logarithmic phase at 37 °C in a 5% carbon dioxide atmosphere using the following culture media containing 10% fetal calf serum (EuroClone, Milan, Italy), antibiotics (50 units/mL penicillin and 50 μg/mL streptomycin) and 2 mM l-glutamine: (i) RPMI-1640 medium (EuroClone, Milan, Italy) for HCT-15, A431, PSN-1, U1285, 2008 and C13* cells; (ii) DMEM (EuroClone, Milan, Italy) for MDA-MB-231 cells.

#### 3.2.2. MTT Assay

The growth inhibitory effect toward tumor cells was evaluated by means of MTT assay. Briefly, 3–8 × 10^3^ cells/well, dependent upon the growth characteristics of the cell line, were seeded in 96-well microplates in growth medium (100 μL). After 24 h, the medium was removed and replaced with a fresh one containing the compound to be studied at the appropriate concentration. Triplicate cultures were established for each treatment. After 72 h, each well was treated with 10 μL of a 5 mg/mL MTT saline solution, and following 5 h of incubation, 100 μL of a sodium dodecyl sulfate (SDS) solution in HCl 0.01 M was added. After overnight incubation, cell growth inhibition was detected by measuring the absorbance of each well at 570 nm using a Bio-Rad 680 microplate reader. Mean absorbance for each drug dose was expressed as a percentage of the control untreated well absorbance and plotted vs. drug concentration. IC_50_ values, the drug concentrations that reduce the mean absorbance at 570 nm to 50% of those in the untreated control wells, were calculated by the four-parameter logistic (4-PL) model. Evaluation was based on means from at least four independent experiments.

#### 3.2.3. Spheroid Cultures

Spheroid cultures were obtained by seeding 2.5 × 10^3^ U1285 cancer cells/well in a round-bottom non-treated tissue culture 96-well plate (Greiner Bio-one, Kremsmünster, Austria) in phenol red free RPMI-1640 medium (Sigma Chemical Co., St. Louis, MO, USA) containing 10% fetal calf serum and supplemented with 20% methyl cellulose stock solution. 

#### 3.2.4. Acid Phosphatase (APH) Assay

An APH modified assay was used for determining cell viability in 3D spheroids, as previously described [67]. Briefly, the pre-seeded spheroids were treated with fresh medium containing the compound to be studied at the appropriate concentration (range 5–150 μM). Triplicate cultures were established for each treatment. After 72 h, each well was treated with 100 μL of the assay buffer (0.1 M sodium acetate, 0.1% Triton-X-100, supplemented with ImmunoPure p-nitrophenyl phosphate; Sigma Chemical Co., St. Louis, MO, USA), and following 3 h of incubation, 10 μL of 1 M NaOH solution was added. The inhibition of the cell growth induced by the tested complexes was detected by measuring the absorbance of each well at 405 nm, using a Bio-Rad 680 microplate reader. Mean absorbance for each drug dose was expressed as a percentage of the control untreated well absorbance (T/C) and plotted vs. drug concentration. IC_50_ values, the drug concentrations that reduce the mean absorbance at 405 nm 50% of those in the untreated control wells, were calculated by four-parameter logistic (4-PL) model. Evaluation was based on means from at least four independent experiments.

#### 3.2.5. Cellular Uptake

U1285 cells (2.5 × 10^6^) were seeded in 75 cm^2^ flasks in growth medium (20 mL). After 24 h, the medium was replaced, and the cells were incubated for 24 h with tested complexes. Monolayers were then washed twice with ice-cold phosphate-buffered saline (PBS), harvested, and counted. Cell samples were subjected to five freeze–thaw cycles at −80 °C and then vigorously vortexed. The samples were treated with highly pure nitric acid (1 mL; Ag < 0.01 μgkg^−1^) and transferred into a microwave Teflon vessel. Samples were then submitted to standard mineralization procedures and analyzed for the silver amount by using a Varian AA Duo graphite furnace atomic absorption spectrometer (Varian, Palo Alto, CA; USA) at 242.795 nm. The calibration curve was obtained using known concentrations of standard solutions purchased from Sigma Chemical Co., St. Louis, MO, USA.

#### 3.2.6. TrxR Inhibition 

Cell-free studies: the assay was performed in PBS buffer pH 7.4, containing 5 mM EDTA, 0.250 mM nicotinamide adenine dinucleotide phosphate (NADPH) and 75 nmol of TrxR1 (Sigma Chemical Co., St. Louis, MO, USA). The silver(I) complexes as well as auranofin were pre-incubated for 5 min at room temperature; the reaction was started with 1 mM DTNB (5,5′-dithiobis(2-nitrobenzoic acid)), and the increase in absorbance was monitored at 412 nm over 5 min at 25 °C. Enzyme activity was calculated taking into account that 1 mol of NADPH yields 2 mol of CNTP anion (carboxy-nitro-thiophenol, reduced DTNB).

Cellular studies: U1285 cells were grown in 75 cm^2^ flasks at confluence and treated with Ag(I) complexes at concentrations corresponding to IC_50_ values for 18 h. At the end of incubation time, cells were collected, washed with ice-cold PBS and centrifuged at ×*g*. Each sample was then lysed with RIPA buffer modified as follows: 150 mM NaCl, 50 mM Tris–HCl, 1% Triton X-100, 1% SDS, 1% DOC, 1 mM NaF, 1 mM EDTA, and immediately before use, an anti-protease cocktail (Roche, Basel, Switzerland) containing PMSF was added. Samples were tested for TrxR activity as above described.

#### 3.2.7. Quantification of Thiols

U1285 cells (2 × 10^5^) were seeded in a six-well plate in growth medium (4 mL). After 24 h, cells were incubated for 36 h with IC_50_ concentrations of tested compounds. Subsequently, the thiol content was measured as previously described [68].

#### 3.2.8. Reactive Oxygen Species Production

The production of ROS was measured in U1285 cells (10^4^ per well) grown for 24 h in a 96-well plate in RPMI medium without phenol red (Sigma Chemical Co., St. Louis, MO, USA). Cells were then washed with ice-cold PBS and loaded with 10 μM 5-(and-6)-chloromethyl-2′,7′-dichlorodihydrofluorescein diacetate acetyl ester (CM–H_2_DCFDA) (Molecular Probes-Invitrogen, Eugene, OR, USA) for 25 min in the dark. Afterward, cells were washed with PBS and incubated with tested compounds. Fluorescence increase was estimated utilizing the wavelengths of 485 (excitation) and 527 nm (emission) in an Infinite^®^ 200 PRO (Tecan, Switzerland) plate reader. Antimycin (3 μM, Sigma Chemical Co., St. Louis, MO, USA), a potent inhibitor of Complex III in the electron transport chain, and auranofin were used as positive controls.

#### 3.2.9. Mitochondrial Membrane Potential (ΔΨ)

The ΔΨ was assayed using the Mito-ID^®^ Membrane Potential Kit according to the manufacturer’s instructions (Enzo Life Sciences, Farmingdale, NY, USA). Briefly, U1285 cells (8 × 10^3^ per well) were seeded in 96-well plates; after 24 h, cells were washed with PBS and loaded with Mito-ID Detection Reagent for 30 min at 37 °C in the dark. Afterward, cells were incubated with increasing concentrations of tested complexes. Fluorescence intensity was estimated using a plate reader (Fluoroskan Ascent FL, Labsystems, Finland) at 490 (excitation) and 590 nm (emission). Carbonyl cyanide m-chlorophenyl hydrazone (CCCP, 4 μM), a chemical inhibitor of the oxidative phosphorylation, was used as a positive control.

#### 3.2.10. TEM Analysis

About 10^6^ U1285 cells were seeded in 24-well plates and, after 24 h incubation, were treated with IC_50_ concentrations of tested compounds and incubated for additional 24 h. Cells were then washed with cold PBS, harvested and directly fixed with 1.5% glutaraldehyde buffer with 0.2 M sodium cacodylate, pH 7.4. After washing with buffer and postfixation with 1% OsO_4_ in 0.2 M cacodylate buffer, specimens were dehydrated and embedded in epoxy resin (Epon Araldite). Sagittal serial sections (1 μm) were counterstained with toluidine blue; thin sections (90 nm) were given contrast by staining with uranyl acetate and lead citrate. Micrographs were taken with a Hitachi H-600 electron microscope (Hitachi, Tokyo, Japan) operating at 75 kV. All photos were typeset in Corel Draw 11.

#### 3.2.11. Cell Death Induction

U1285 cells were seeded into 8-well tissue-culture slides (BD Falcon, Bedford, MA, USA) at 5 × 10^4^ cells/well (0.8 cm^2^). After 24 h, the cells were washed twice with PBS, and following 48 h of treatment with IC_50_ doses of the tested compound, cells were stained for 5 min with 10 µg/mL of Hoechst 33258 (20-(4-hydroxyphenyl)-5-(4-methyl-1-piperazinyl)-2,50-bi-1H-benzimidazole trihydrochloride hydrate, Sigma-Aldrich, St. Louis, MI, USA) in PBS. Samples were examined at 5× and 40× magnification in a Zeiss LSM 800 confocal microscope using the Zeiss ZEN 2.3 software system.

#### 3.2.12. Statistical Analysis

All values are the means ± v SD of no less than three measurements starting from three different cell cultures. Multiple comparisons were made by ANOVA followed by the Tukey–Kramer multiple comparison test (* *p* < 0.05, ** *p* < 0.01), using GraphPad InStat software (GraphPad Software, San Diego, CA, USA).

## 4. Conclusions

The methyl ester derivatives L^OMe^ and L^2OMe^ of bis(pyrazol-1-yl)- and bis(3,5-dimethyl-pyrazol-1-yl)-acetic acid were synthesized and used for the preparation of the silver(I) complexes **3**–**5** following a one-pot synthesis in methanol. The lipophilic PPh_3_ and the hydrophilic PTA were selected as coligands to stabilize silver in +1 oxidation state. The compounds were fully characterized both in solid state and in solution.

All the investigated complexes showed significant cytotoxic effects against a panel of human cancer cell lines, with IC_50_ values in the low/sub-micromolar range, and proved to be more effective than the reference metallodrug cisplatin, especially when tested against the highly aggressive and intrinsically resistant SCLC cells, either in 2D or 3D cancer cell models. Mechanistic investigations of the cytotoxic effects demonstrated that the newly developed Ag(I) complexes induce an oxidative shift in the redox status of SCLC cells as well as a direct antimitochondrial effect due to their ability to target cellular TrxR. These results, besides supporting the hypothesis that TrxR represents a major cellular target for this class of complexes, suggest that TrxR inhibition by redox modulators, such as Ag(I) complexes, could represent a valuable strategy to target intrinsically refractory SCLC cells. 

## Figures and Tables

**Table 1:** Cytotoxicity of ligands **1** and **2**, coligands PPh_3_ and PTA, newly developed silver(I) complexes **3**–**5** and cisplatin on 2D cell cultures.

	IC_50_ (µM) ± S.D.							
	HCT-15	PSN-1	A431	U1285	MDA-MB-231	2008	C13* (R.F.)	HEK293
**1**	>50	>50	>50	>50	>50	>50	>50	>50
**2**	>50	>50	>50	>50	>50	>50	>50	>50
**3**	6.5 ± 0.5	24.6 ± 0.9	4.8 ± 1.8	2.1 ± 0.3	5.4 ± 0.9	6.9 ± 1.2	5.7 ± 0.2 (0.8)	17.5 ± 1.3
**4**	9.2 ± 2.9	1.2 ± 0.4	0.9 ± 0.2	0.7 ± 0.1	10.6 ± 1.9	1.3 ± 0.2	0.20 ± 0.02 (0.2)	15.7 ± 1.8
**5**	7.4 ± 0.9	3.7 ± 0.4	1.4 ± 0.4	1.2 ± 0.2	2.8 ± 0.4	1.4 ± 0.3	0.5 ± 0.1 (0.4)	13.8 ± 2.1
**PPh_3_**	>50	>50	47.5 ± 1.1	>50	>50	>50	>50	>100
**PTA**	>100	>100	>100	>100	>100	>100	>100	>100
**cisplatin**	15.3 ± 2.6	18.3 ± 3.1	2.1 ± 0.9	9.6 ± 1.2	30.5 ± 2.6	2.2 ± 1.0	30.5 ± 2.6 (13.9)	21.6 ± 3.5

Cells (3 − 8 × 10^3^ × mL^−1^) were treated for 72 h with increasing concentrations of tested compounds. Cytotoxicity was assessed by MTT test. The IC_50_ values were calculated by the four-parameter logistic model (*p* < 0.05). R.F. = IC_50_ (resistant subline)/IC_50_ (wild-type cells). S.D. is standard deviation.

**Table 2:** Cytotoxicity of ligands **1** and **2**, Ag(I) complexes **3**–**5** and cisplatin toward U1285 cancer cell spheroids.

	IC_50_ (µM) ± S.D.
**1**	>100
**2**	>100
**3**	63.8 ± 4.4
**4**	27.9 ± 1.8
**5**	22.0 ± 2.1
**cisplatin**	65.4 ± 1.4

## Data Availability

The data presented in this study are available from the authors on request.

## Supplementary Materials

The supporting information can be downloaded at: https://www.mdpi.com/article/10.3390/ijms24044091/s1.
