# Supplementary material for: Novel Silver Complexes Based on Phosphanes and Ester Derivatives of Bis(pyrazol-1-yl)acetate Ligands Targeting TrxR: New Promising Chemotherapeutic Tools Relevant to SCLC Management
Source: Int J Mol Sci. 2023 Feb 17;24(4):4091. doi: 10.3390/ijms24044091 (PMC9960633; doi:10.3390/ijms24044091)
Supplement: Supplementary file 1 [file ijms-24-04091-s001.zip › ijms-2208881-supplementary.pdf]

International Journal of *Molecular Sciences*

MDPI

Supplementary material

Novel Silver Complexes Based on Phosphanes and Ester Derivatives of Bis(pyrazol-1-yl)acetate Ligands Targeting TrxR: New Promising Chemotherapeutic Tools Relevant to SCLC Management

Maura Pellei, Carlo Santini, Luca Bagnarelli, Miriam Caviglia, Paolo Sgarbossa, Michele De Franco, Mirella Zancato, Cristina Marzano and Valentina Gandin

Table of Contents:

Figure S1. Correlation between cytotoxicity and cellular uptake (panel **A**) and between cellular uptake and logP of phosphane co-ligands (panel **B**) in treated U1285 cancer cells.
Figure S2: ^{1}H-NMR spectrum of [HC(pz)$_2$COOCH$_3$] (L^{OMe}, **1**) in CD$_3$CN.
Figure S3: ^{1}H-NMR spectrum of [HC(pz^{Me2})$_2$COOCH$_3$] (L^{2OMe}, **2**) in CD$_3$CN.
Figure S4: FT-IR spectra of [Ag(PPh$_3$)(L^{OMe})]NO$_3$ (**3**).
Figure S5: ^{1}H-NMR spectrum of [Ag(PPh$_3$)(L^{OMe})]NO$_3$ (**3**) in CD$_3$CN.
Figure S6: ^{13}C{^{1}H}-NMR spectrum of [Ag(PPh$_3$)(L^{OMe})]NO$_3$ (**3**) in CD$_3$CN.
Figure S7: ^{31}P{^{1}H}-NMR spectrum of [Ag(PPh$_3$)(L^{OMe})]NO$_3$ (**3**) in CD$_3$CN at 243 K.
Figure S8: ^{31}P{^{1}H}-NMR spectrum of [Ag(PPh$_3$)(L^{OMe})]NO$_3$ (**3**) in CD$_3$OD at 223 K.
Figure S9: FT-IR spectra of [Ag(PPh$_3$)(L^{2OMe})]NO$_3$ (**4**).
Figure S10: ^{1}H-NMR spectrum of [Ag(PPh$_3$)(L^{2OMe})]NO$_3$ (**4**) in CD$_3$CN.
Figure S11: ^{13}C{^{1}H}-NMR spectrum of [Ag(PPh$_3$)(L^{2OMe})]NO$_3$ (**4**) in CD$_3$CN.
Figure S12: ^{31}P{^{1}H}-NMR spectrum of [Ag(PPh$_3$)Ag(L^{2OMe})]NO$_3$ (**4**) in CDCN$_3$ at 243 K.
Figure S13: ^{31}P{^{1}H}-NMR spectrum of [Ag(PPh$_3$)(L^{2OMe})]NO$_3$ (**4**) in CDCl$_3$ at 223 K.
Figure S14: FT-IR spectra of [Ag(PTA)(L^{2OMe})]NO$_3$ (**5**).
Figure S15: ^{1}H-NMR spectrum of [Ag(PTA)(L^{2OMe})]NO$_3$ (**5**) in CD$_3$CN.
Figure S16: ^{13}C{^{1}H}-NMR spectrum of [Ag(PTA)(L^{2OMe})]NO$_3$ (**5**) in CD$_3$CN.
Figure S17: ^{31}P{^{1}H}-NMR spectrum of [Ag(PTA)(L^{2OMe})]NO$_3$ (**5**) in CD$_3$CN at 243 K.

A

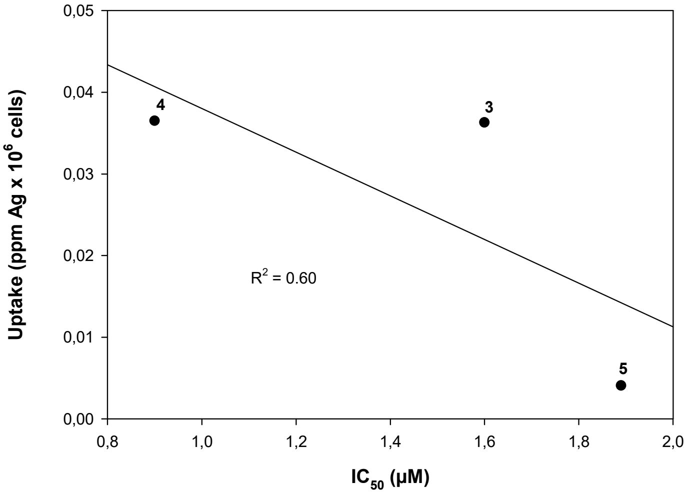

B

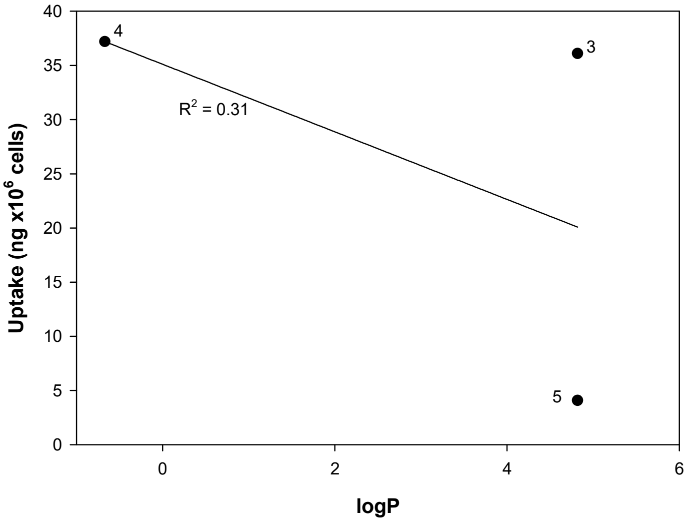

Figure S1. Correlation between cytotoxicity and cellular uptake (panel **A**) and between cellular uptake and logP of phosphane co-ligands (panel **B**) in treated U1285 cancer cells.

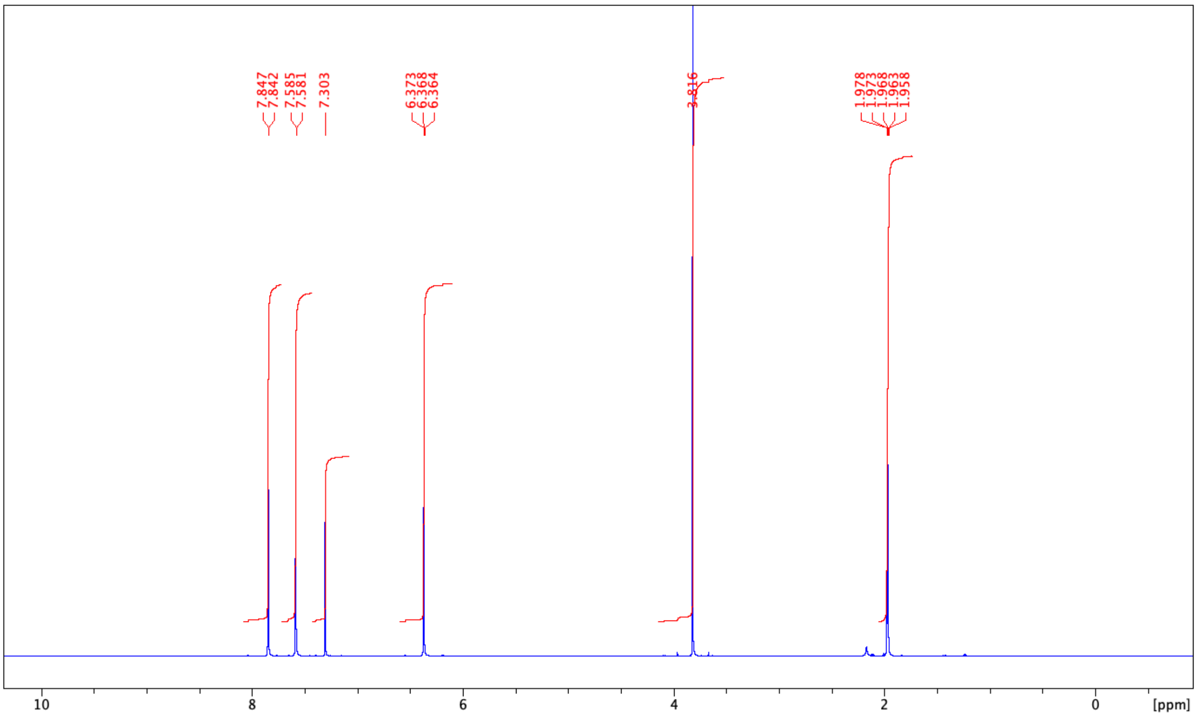

Figure S2. ^{1}H-NMR spectrum of [HC(pz)$_2$COOCH$_3$] (L^{OMe}, **1**) in CD$_3$CN.

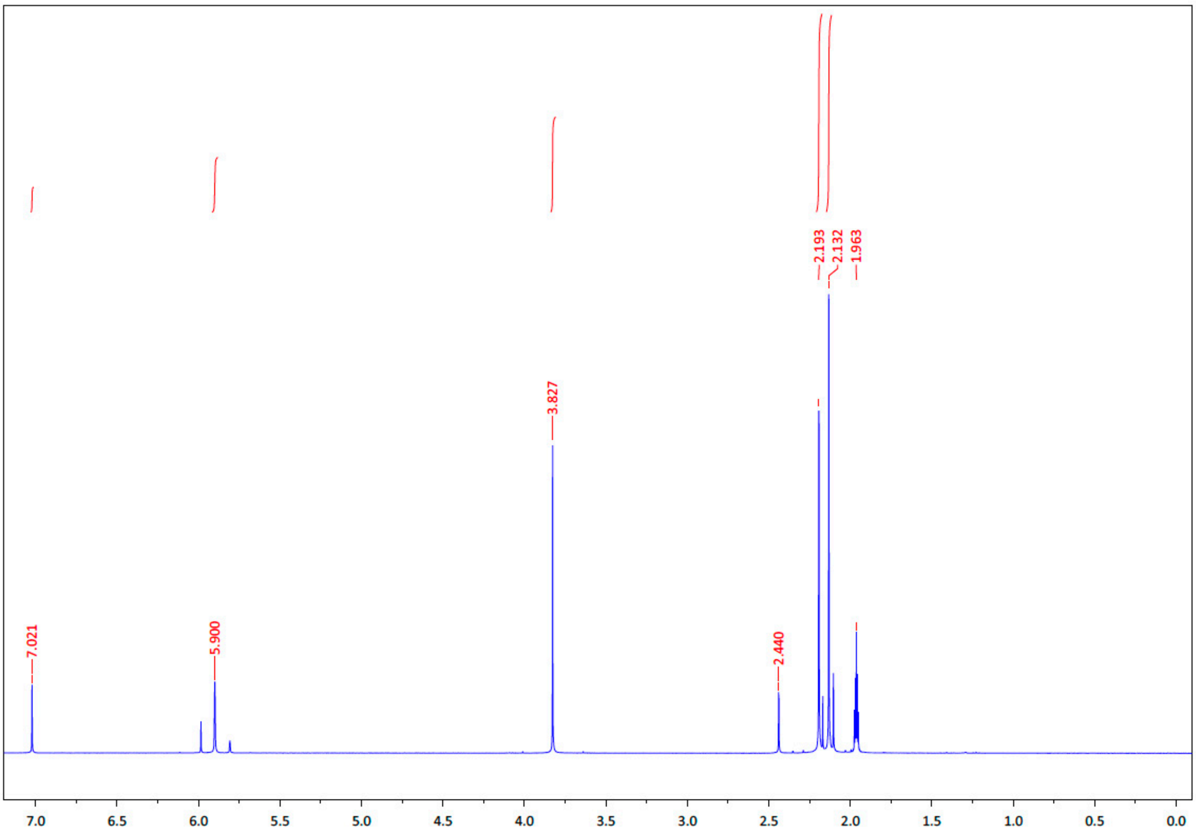

Figure S3. ^{1}H-NMR spectrum of [HC(pz^{Me2})$_2$COOCH$_3$] (L^{2OMe}, **2**) in CD$_3$CN.

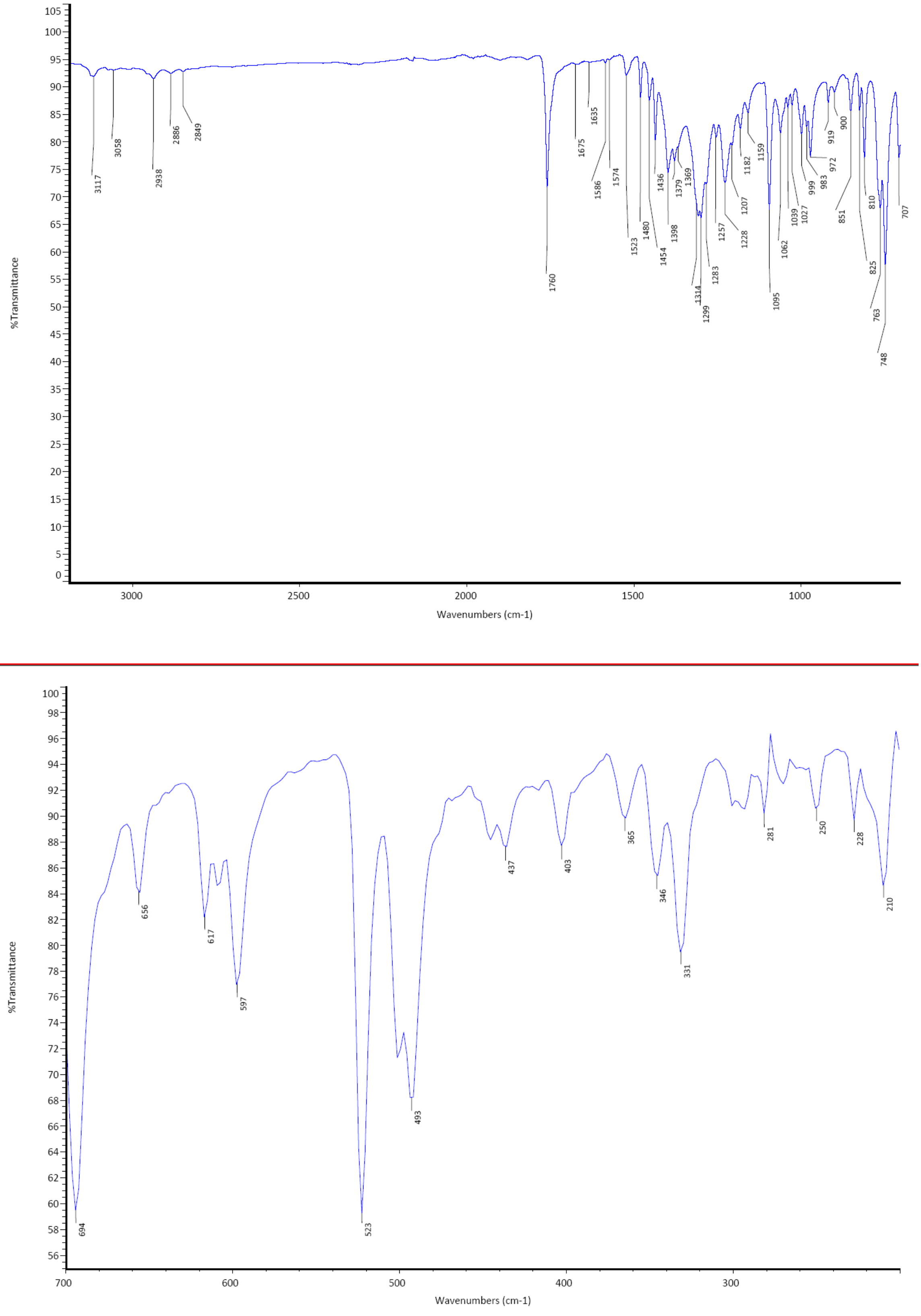

Figure S4. FT-IR spectra of [Ag(PPh₃)(L^OMe^)]NO₃ (**3**).

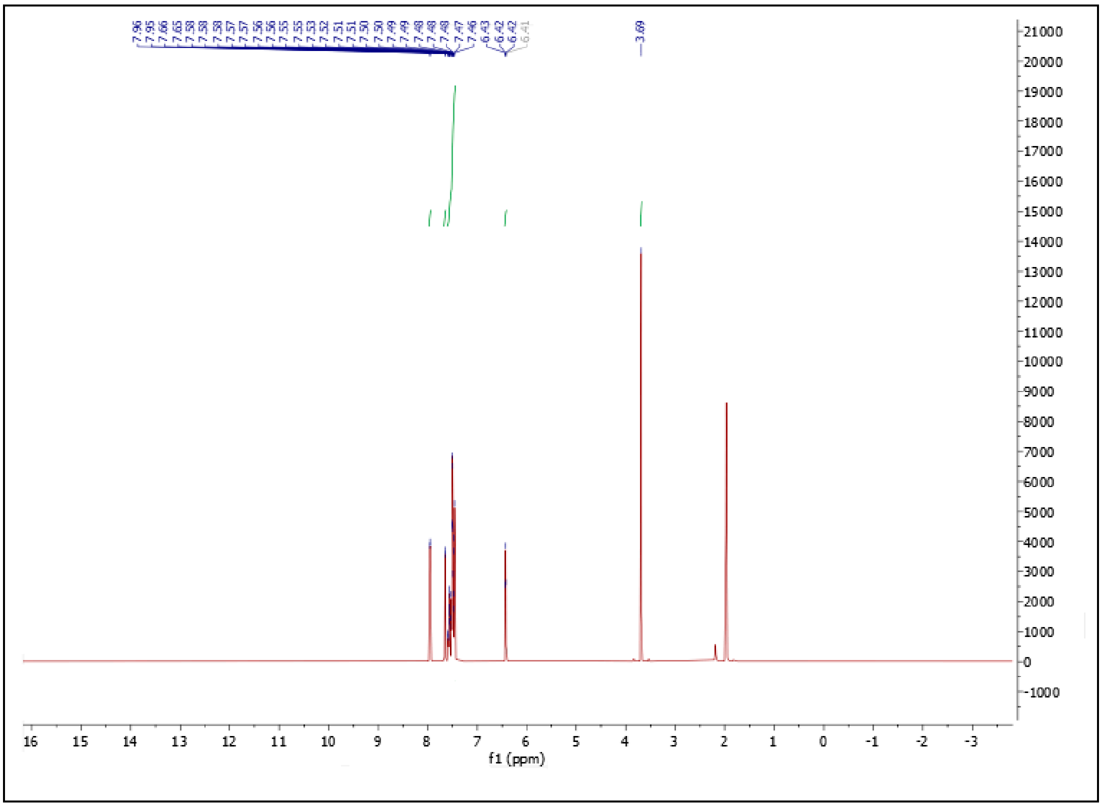

Figure S5. ^{1}H-NMR spectrum of $[Ag(PPh_3)(L^{OMe})]NO_3$ (**3**) in CD_3CN.

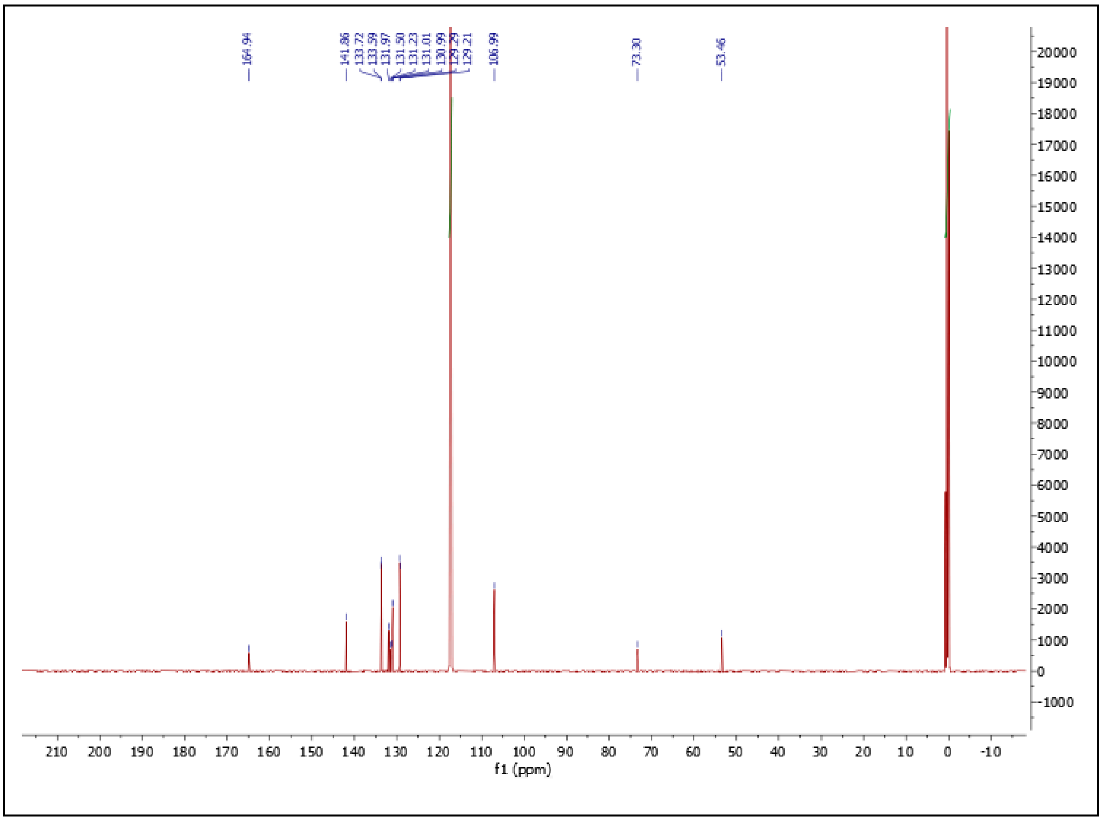

Figure S6. $^{13}C\{^1H\}$-NMR spectrum of $[Ag(PPh_3)(L^{OMe})]NO_3$ (**3**) in CD_3CN.

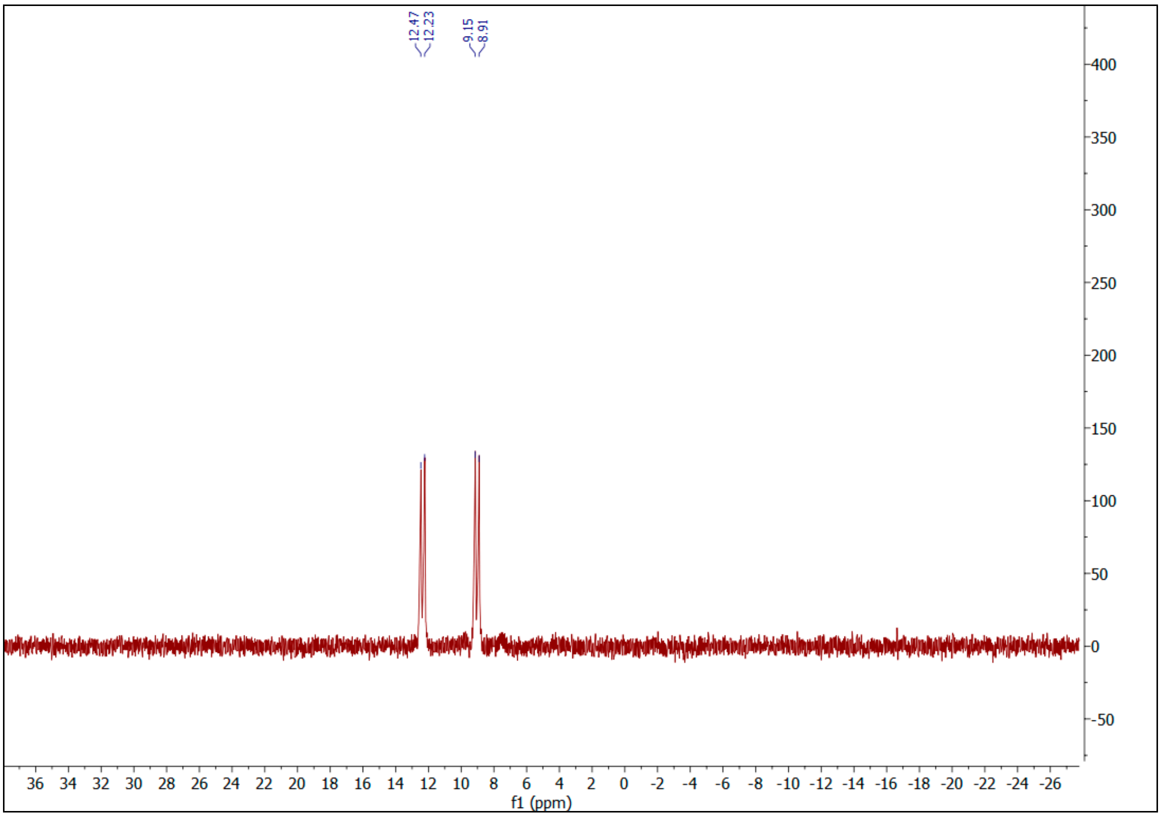

Figure S7. $^{31}P\{^1H\}$-NMR spectrum of $[Ag(PPh_3)(L^{OMe})]NO_3$ (**3**) in CD_3CN at 243 K.

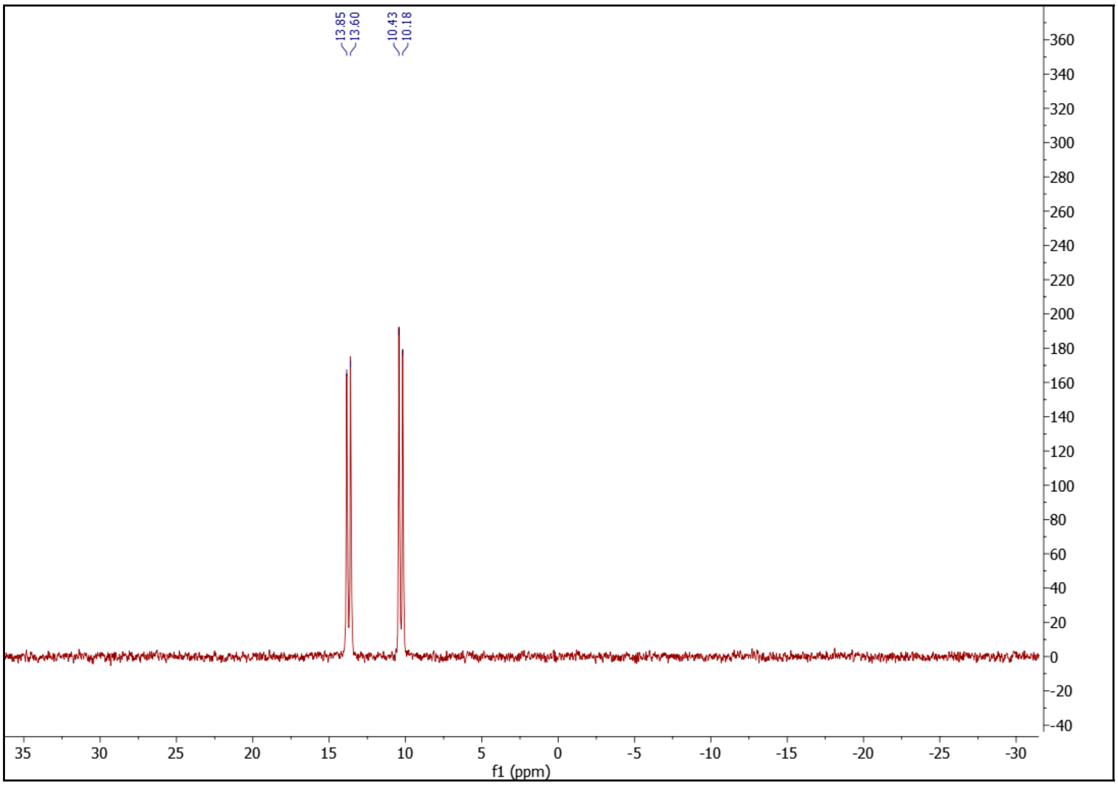

Figure S8. $^{31}P\{^1H\}$-NMR spectrum of $[Ag(PPh_3)(L^{OMe})]NO_3$ (**3**) in CD_3OD at 223 K.

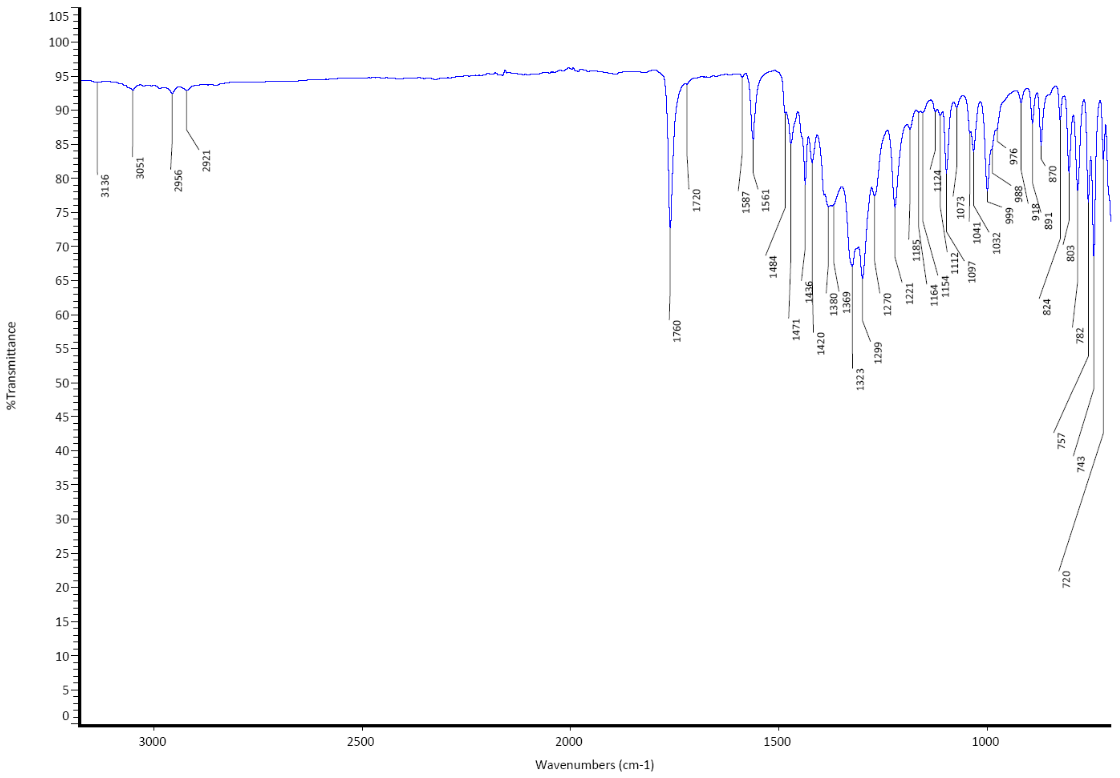

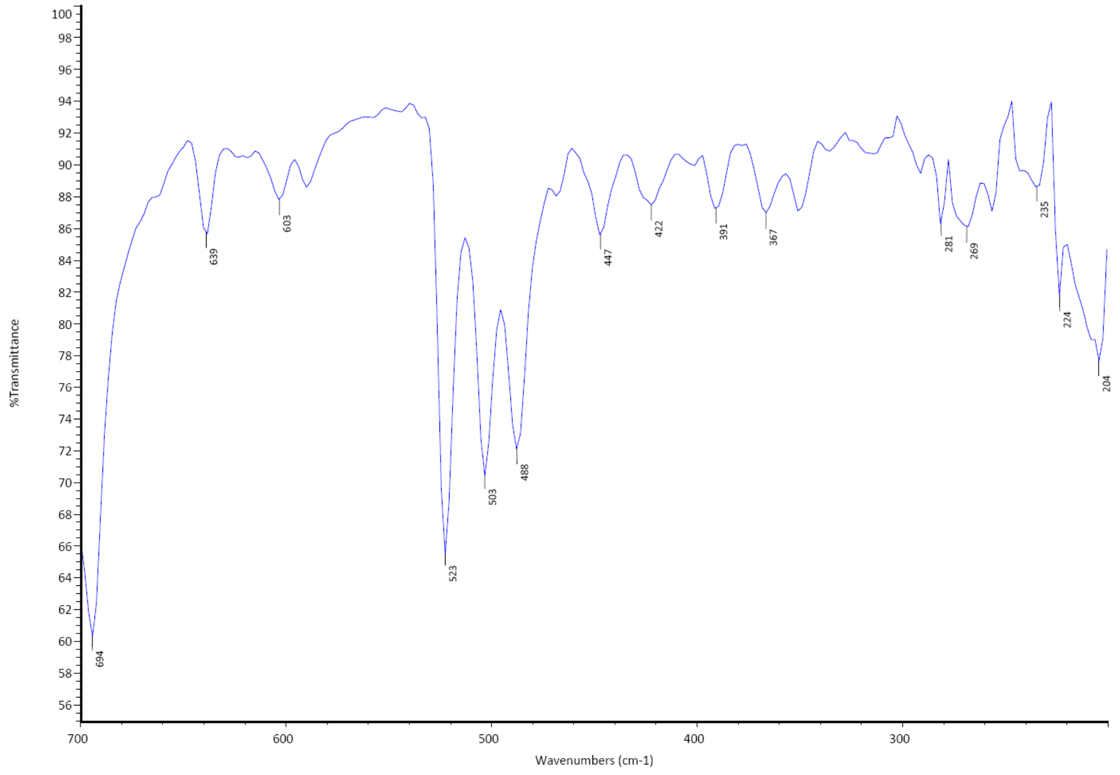

Figure S9. FT-IR spectra of [Ag(PPh3)(L^{2OMe})]NO3 (**4**).

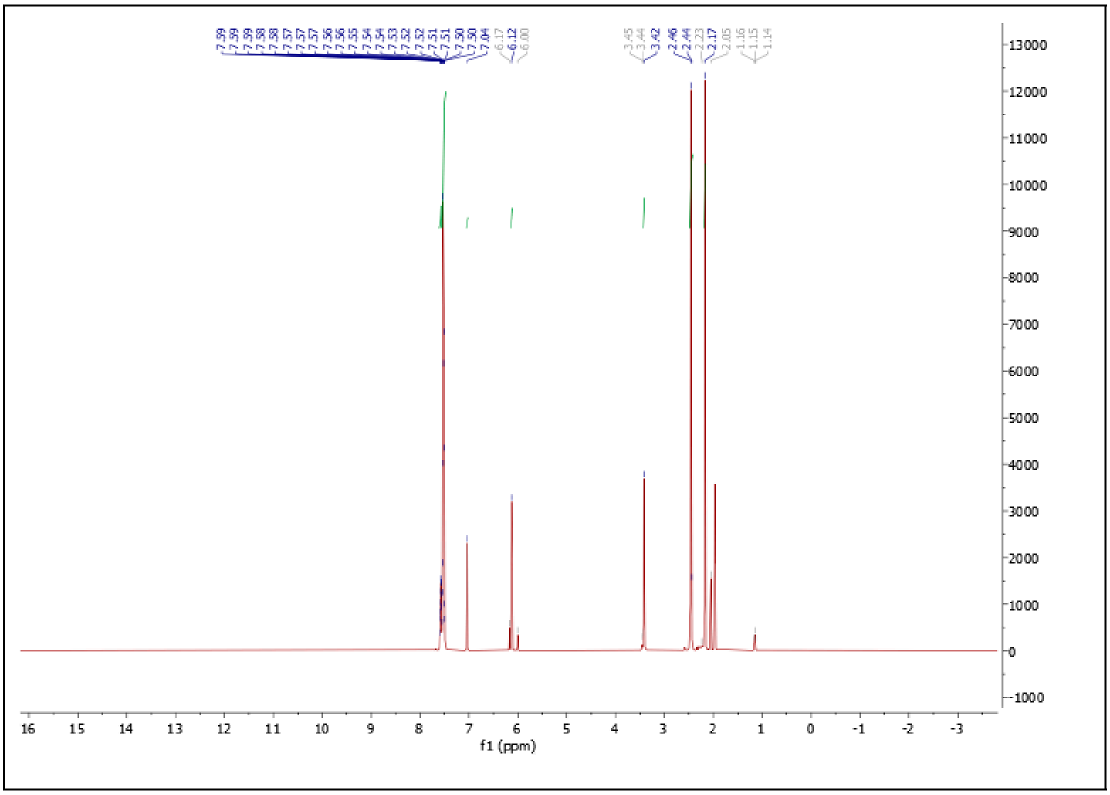

Figure S10. ^{1}H-NMR spectrum of $[Ag(PPh_3)(L^{2OMe})]NO_3$ (**4**) in CD_3CN.

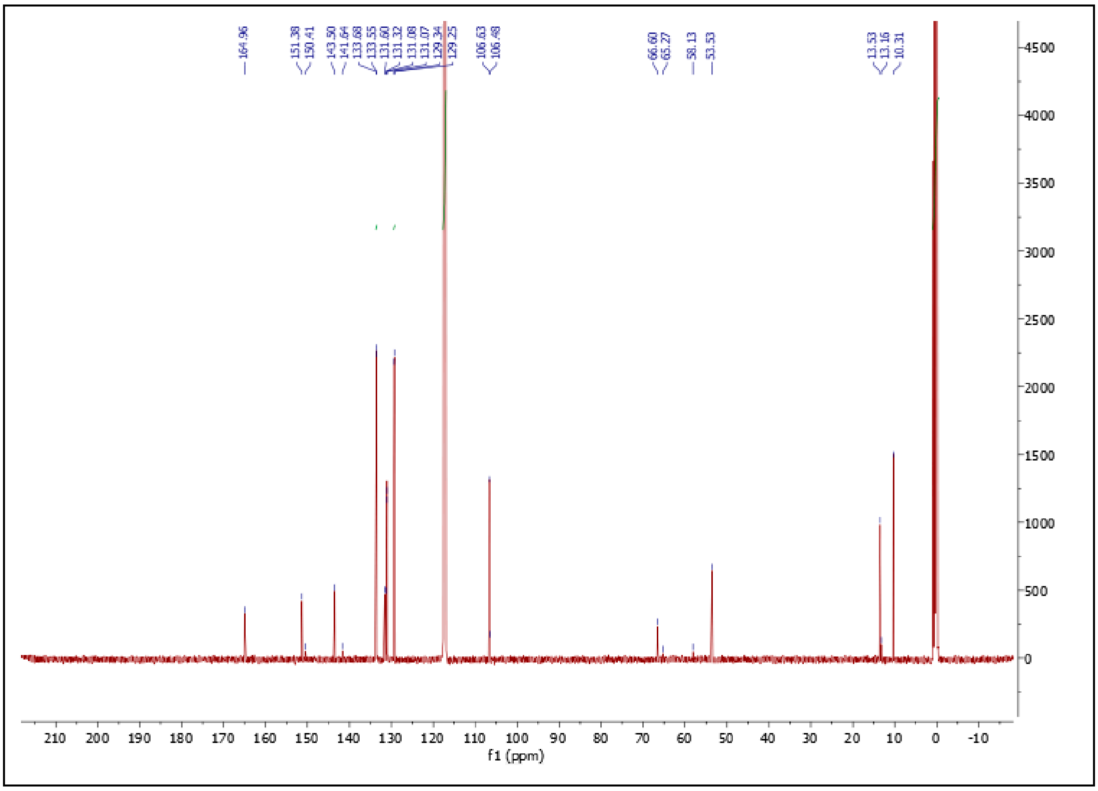

Figure S11. $^{13}C\{^{1}H\}$-NMR spectrum of $[Ag(PPh_3)(L^{2OMe})]NO_3$ (**4**) in CD_3CN.

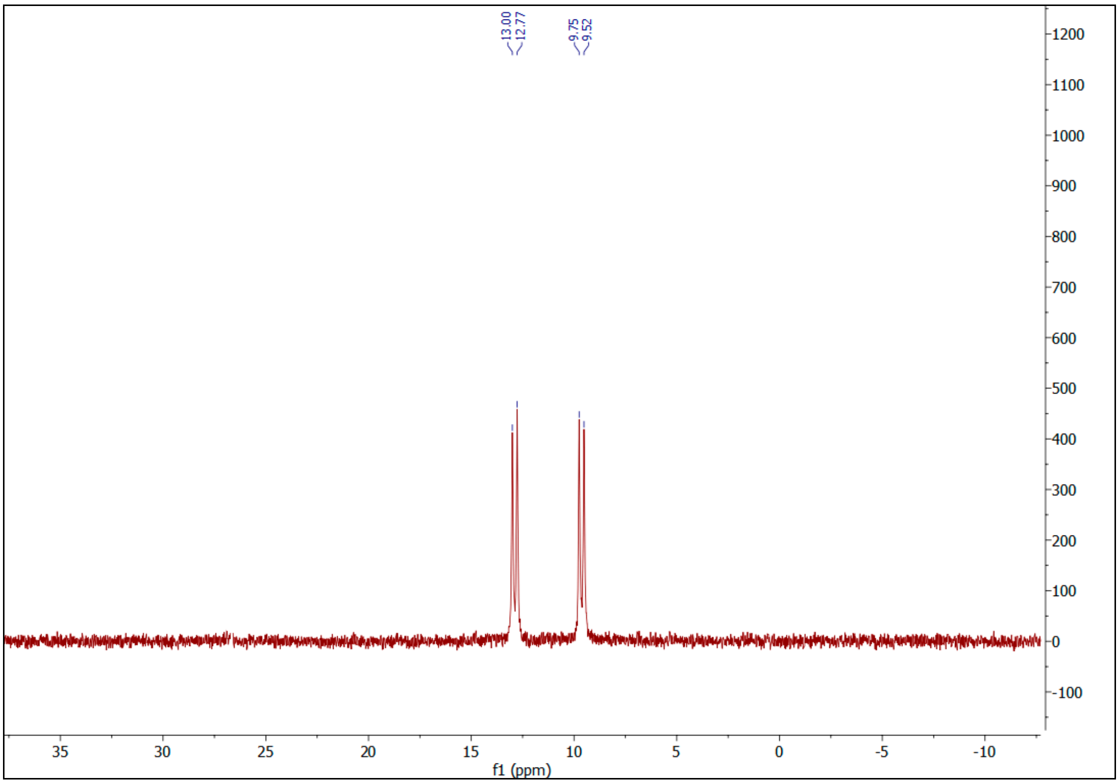

Figure S12. $^{31}P\{^1H\}$-NMR spectrum of $[Ag(PPh_3)(L^{2OMe})]NO_3$ (**4**) in CD_3CN at 243 K.

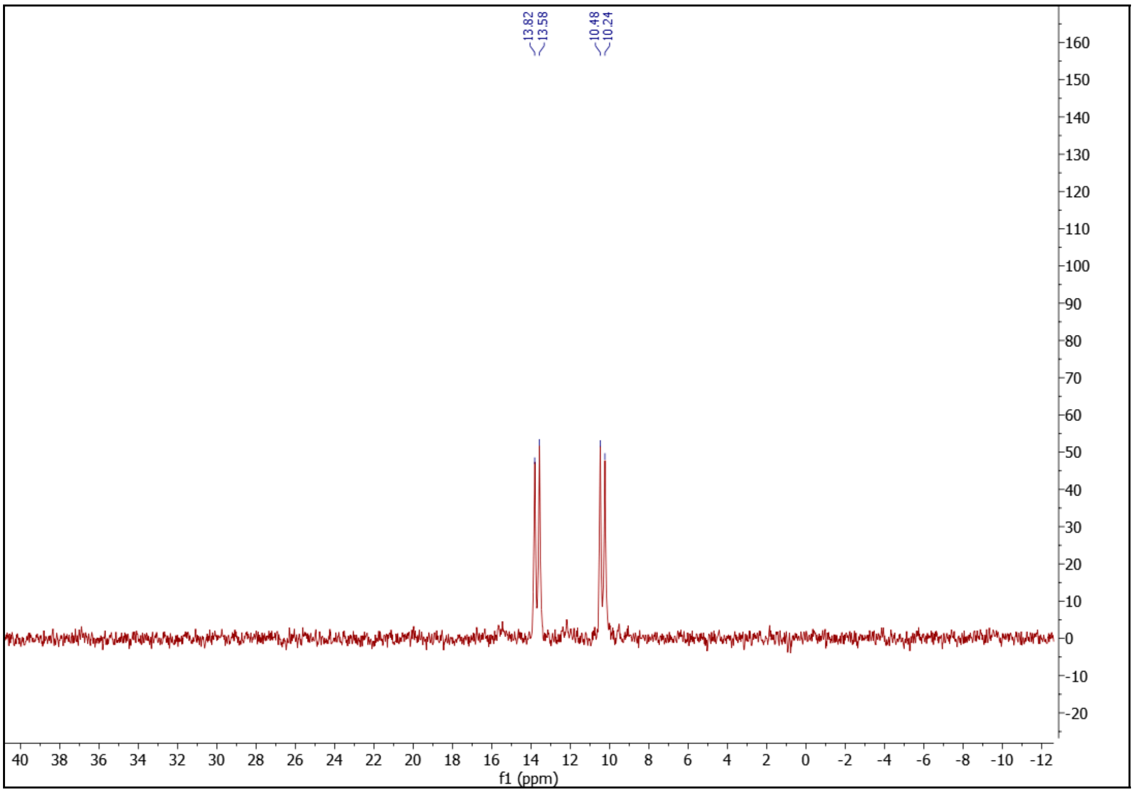

Figure S13. $^{31}P\{^1H\}$-NMR spectrum of $[Ag(PPh_3)(L^{2OMe})]NO_3$ (**4**) in $CDCl_3$ at 223 K.

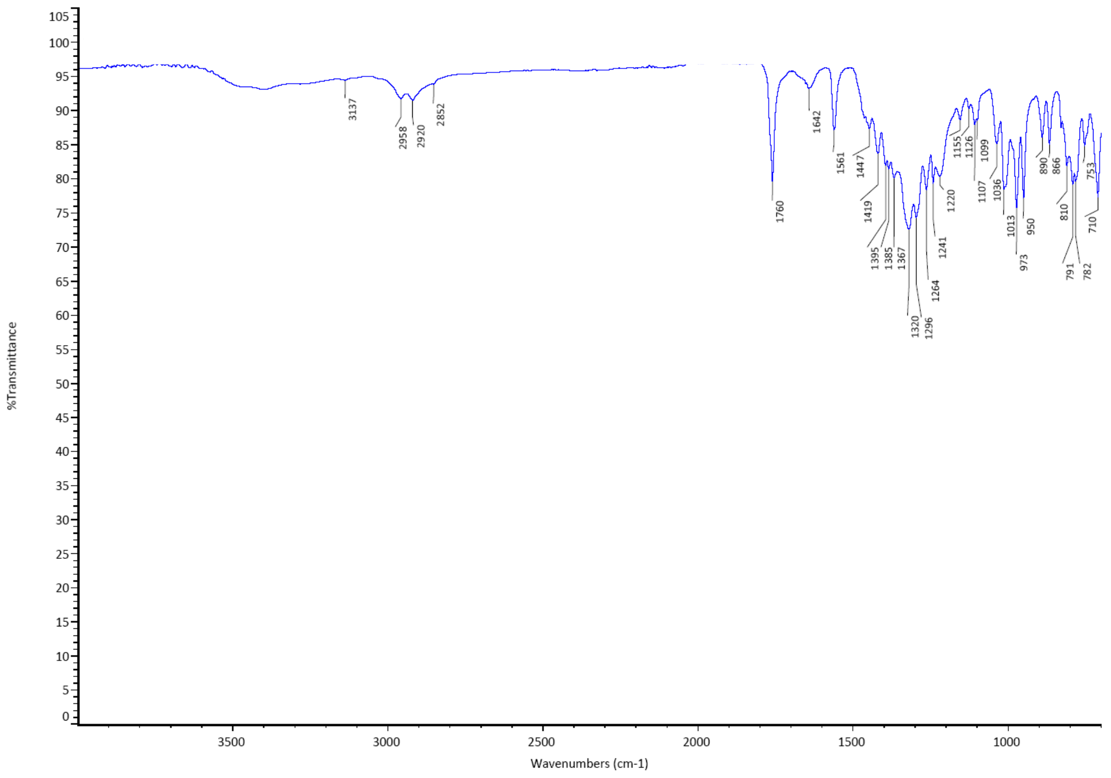

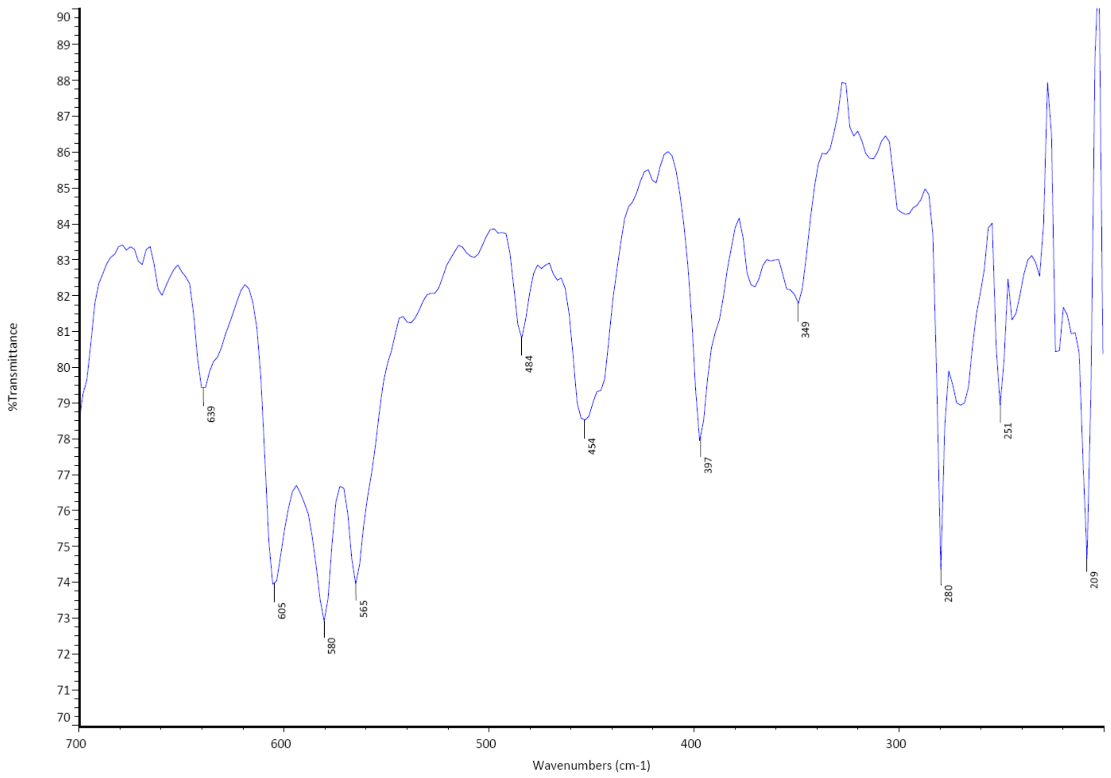

Figure S14. FT-IR spectra of [Ag(PTA)(L^{2OMe})]NO_3 (**5**).

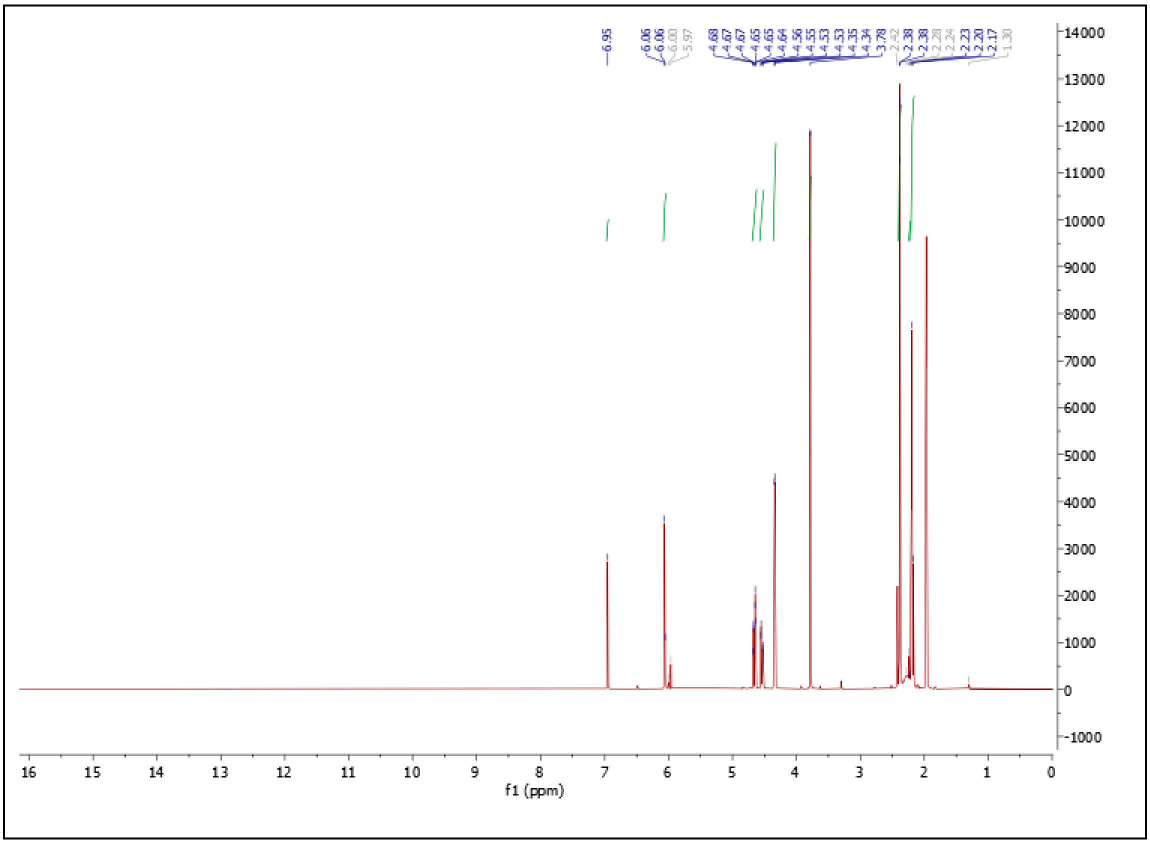

Figure S15. ^{1}H-NMR spectrum of [Ag(PTA)(L^{2OMe})]NO_3 (**5**) in CD_3CN.

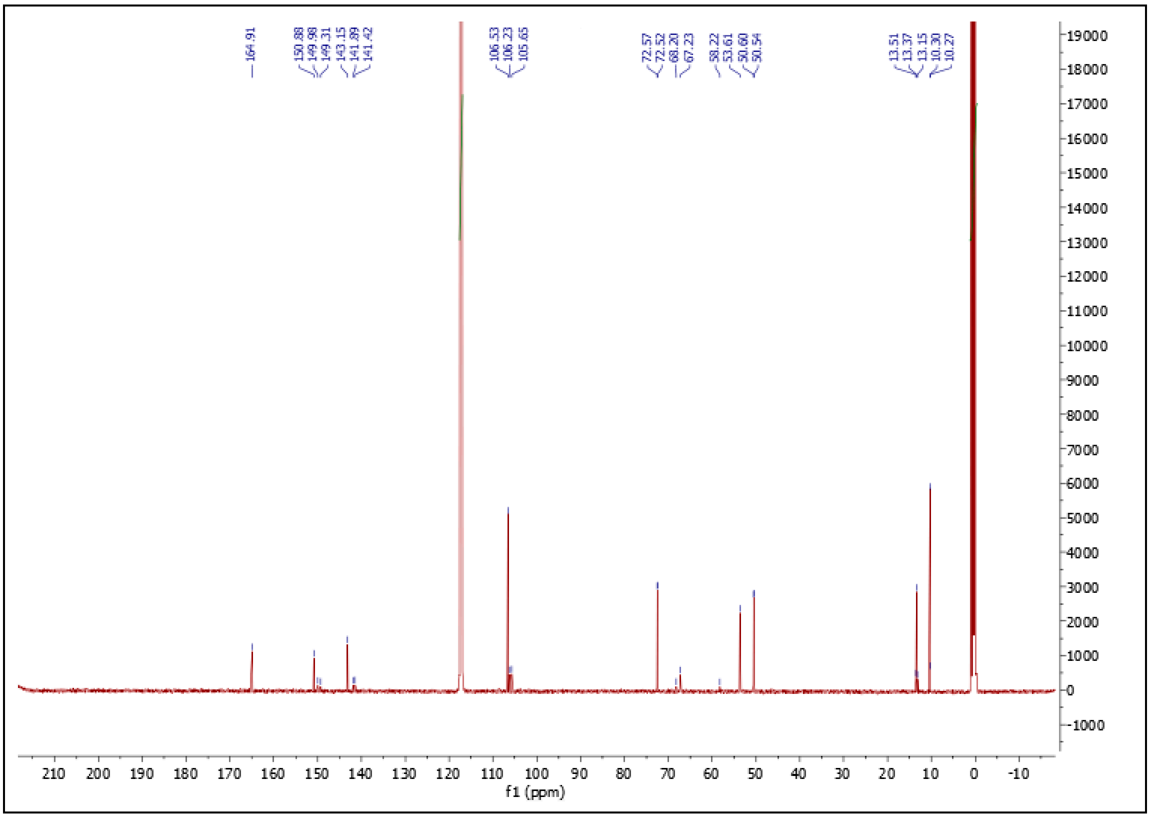

Figure S16. ^{13}C{^{1}H}-NMR spectrum of [Ag(PTA)(L^{2OMe})]NO_3 (**5**) in CD_3CN.

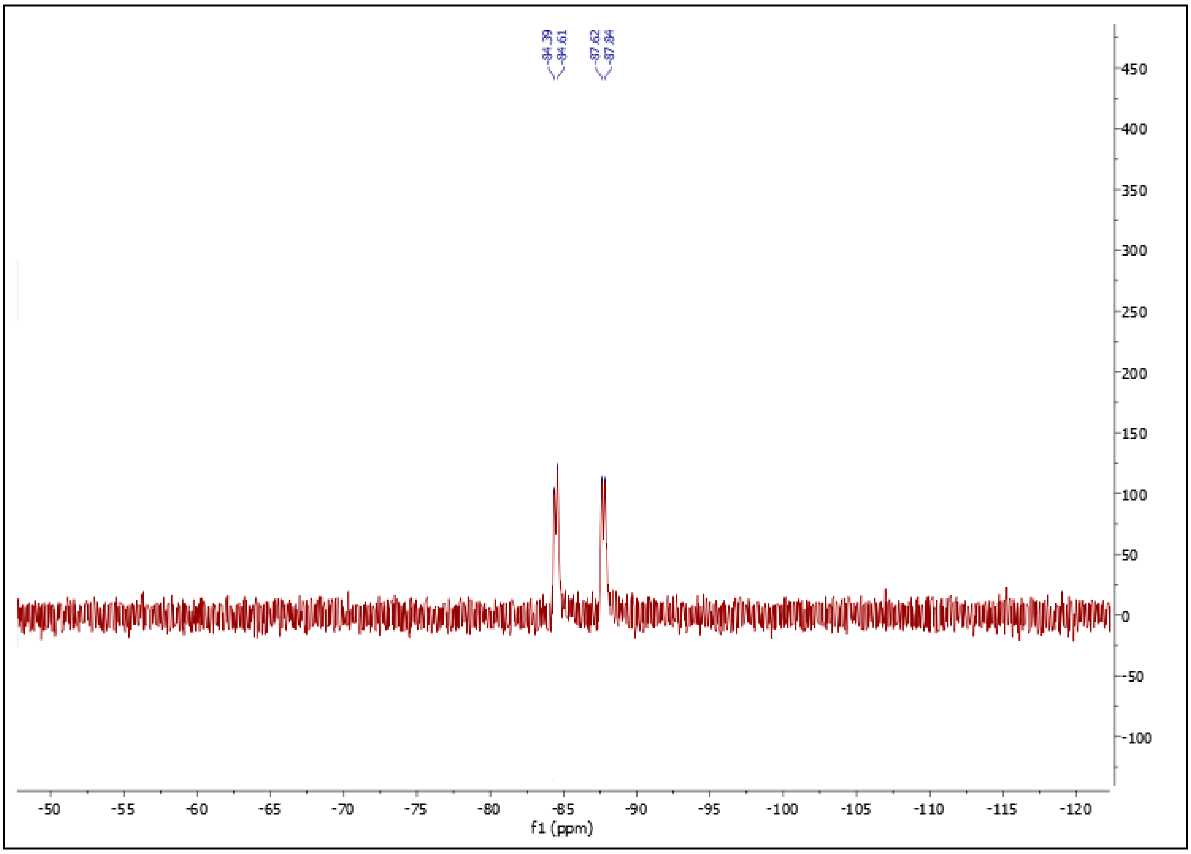

Figure S17. $^{31}P\{^{1}H\}$-NMR spectrum of $[Ag(PTA)(L^{2OMe})]NO_3$ (**5**) in CD_3CN at 243 K.
